# Structure based aggregation studies reveal the presence of helix-rich intermediate during α-Synuclein aggregation

**DOI:** 10.1038/srep09228

**Published:** 2015-03-18

**Authors:** Dhiman Ghosh, Pradeep K. Singh, Shruti Sahay, Narendra Nath Jha, Reeba S. Jacob, Shamik Sen, Ashutosh Kumar, Roland Riek, Samir K. Maji

**Affiliations:** 1 Department of Biosciences and Bioengineering, IIT Bombay, Powai, Mumbai, India 400076; 2 Laboratory for Physical Chemistry, Wolfgang-Pauli-Str. 10, ETH Zurich, CH-8093 Zurich, Switzerland

## Abstract

Mechanistic understanding of nucleation dependent polymerization by α-synuclein (α-Syn) into toxic oligomers and amyloids is important for the drug development against Parkinson's disease. However the structural and morphological characterization during nucleation and subsequent fibrillation process of α-Syn is not clearly understood. Using a variety of complementary biophysical techniques monitoring entire pathway of nine different synucleins, we found that transition of unstructured conformation into β-sheet rich fibril formation involves helix-rich intermediates. These intermediates are common for all aggregating synucleins, contain high solvent-exposed hydrophobic surfaces, are cytotoxic to SHSY-5Y cells and accelerate α-Syn aggregation efficiently. A multidimensional NMR study characterizing the intermediate accompanied with site-specific fluorescence study suggests that the N-terminal and central portions mainly participate in the helix-rich intermediate formation while the C-terminus remained in an extended conformation. However, significant conformational transitions occur at the middle and at the C-terminus during helix to β-sheet transition as evident from Trp fluorescence study. Since partial helix-rich intermediates were also observed for other amyloidogenic proteins such as Aβ and IAPP, we hypothesize that this class of intermediates may be one of the important intermediates for amyloid formation pathway by many natively unstructured protein/peptides and represent a potential target for drug development against amyloid diseases.

α-Synuclein (α-Syn) is a 140 amino acid soluble protein, which is abundantly expressed in brain^1^. Although α-Syn is present in almost all the sub cellular pools of neurons, it is mostly localized in the presynaptic terminals and nucleus^2^,^3^. The exact physiological function of α-Syn is not clearly understood, however, several studies have shown the involvement of α-Syn in regulation of presynaptic vesicle pool, neurotransmitter release, synaptic function as well as neuronal plasticity^4^,^5^. In contrast, α-Syn has been shown to play a central role in the pathogenesis of Parkinson's disease (PD), a very common neurodegenerative disorder in aged humans^6^. Aggregated α-Syn is the major component of the Lewy bodies (LBs) and Lewy neurites (LNs) in the brain of patients – the major pathological hallmarks of PD^7^. The discovery of several autosomal dominant missense mutations as well as duplication and triplication of gene encoding α-Syn are associated with rare familial forms of PD, further supporting that α-Syn aggregation is linked to PD pathogenesis^8^,^9^,^10^,^11^,^12^,^13^,^14^,^15^,^16^. Further, overexpression of human α-Syn and its familial PD associated mutants in neuronal cells as well as in various animal models results in toxicity and recapitulation of many symptoms of PD^17^,^18^.

Several *in vitro* studies have revealed that α-Syn is natively unfolded protein under physiological conditions and can self-assemble into highly ordered amyloid fibrils upon incubation for a longer time^19^. The primary sequence of α-Syn helps it to adopt many distinct local or global conformations. The N-terminus (1–60 residues) of α-Syn has seven imperfect repeats (with conserved KTKEGV motif) characteristic of an amphipathic helix of apolipoproteins^1^,^20^. The middle hydrophobic region of α-Syn (61–95 residues) has a high propensity for β-sheet rich secondary structure and known to drive the amyloid formation by the protein^20^,^21^. The negatively charged C-terminus (96–140 residues) has no secondary structure preference and is highly disordered and shown to regulate the oligomerization and aggregation of α-Syn^22^.

α-Syn at the presynaptic terminals has shown to be associated with the synaptic vesicles and this vesicle association is important for its vesicular transport from cell body to synaptic terminals^23^. It was suggested that alteration in the vesicle/membrane binding ability of α-Syn might result in pathogenesis. However, there are reports, which also suggest that aggregation ability of α-Syn in the vicinity of membranes is a major contributing factor for compromised membrane integrity associated with α-Syn pathogenesis. For example, it has been recently shown that despite retaining the membrane binding affinity, an aggregation-defective α-Syn mutant was unable to initiate damage in supported lipid bilayers^24^. However, amyloid-mediated membrane disruption is a complex phenomenon and this multistep cascade can be modulated by several factors, including peptide topology in the membrane, presence of cationic switches and degree of curvatures in membrane^25^,^26^,^27^,^28^. *In vitro* studies also suggest that α-Syn binds strongly to negatively charged membrane vesicles mostly through N-terminal ~1–100 residues and adopt α-helical structure^22^. It has been also found that N-terminus is not only important for monomer-membrane interaction, however, also crucial for oligomer-membrane interactions^29^. Aggregation studies of α-Syn in presence of membranes/lipids have suggested that moderate membrane binding of α-Syn facilitates its aggregation. Along with this observation, the partial helix promotion by 2, 2, 2-trifluoroethanol (moderate concentration (v/v)) and sodium dodecyl sulphate (SDS) also accelerates the amyloid formation of α-Syn^30^,^31^. The membrane-mimicking detergent micelles has been used to characterize the three-dimensional NMR structure of a stable helical intermediate of IAPP, involved in type II diabetes^32^. Moreover, using SDS as a model solvent it also has been demonstrated that α-Syn folding involves multistates and in this lipid mimetic environment (SDS) it adopts a partially folded thermodynamic intermediate with helix-rich conformation^33^. However, amyloid aggregation process in solution and on membrane is quite different and might involve different partially folded intermediates^34^,^35^. Therefore, elucidating the three -dimensional structures of these oligomeric species and their membrane/ligand interaction could be helpful for potential therapeutics against neurodegenerative disorders^36^,^37^,^38^,^39^.

Previously it was shown that one of the intermediates α-Syn aggregation possesses α-helical conformation^40^ raising the possibility that α-Syn aggregation may involve helix-rich intermediate. The helical intermediates also shown to appear during the aggregation of the Aβ protein^41^ associated with Alzheimer's disease and IAPP aggregation in type II diabetes (both in presence and absence of glycosaminoglycan)^42^,^43^,^44^,^45^. Recently, it is reported that (IAPP) forms soluble helix-rich oligomers that induce apoptosis in cultured pancreatic cells^44^. Based on these findings, it was suggested that natively unstructured protein/peptides might form toxic helical intermediate during their amyloid fibril assembly^41^,^42^,^46^,^47^,^48^,^49^,^50^, which could be targeted for the drug development against these amyloid diseases^51^.

To study the structural and morphological transformation by α-Syn during entire pathway of its aggregation, we monitored *in vitro* α-Syn aggregation by time-dependent CD spectroscopy (for structural transition) along with ThT fluorescence (monitoring amyloid kinetics) and morphological transformation by AFM. We also included familial PD associated mutants of α-Syn (A30P, E46K and A53T), a designed mutant E57K as well as other members of synuclein family (β- and γ-synuclein) in our aggregation studies. We showed that all aggregating synuclein formed oligomeric helix-rich state at the beginning of elongation phase before converting to the β-sheet rich matured fibrils. This helix-rich state is the heterogeneous mixture of various α-Syn species including helical oligomers, β-sheet rich fibrils and soluble unstructured α-Syn. The helix-rich states are more cytotoxic compared to higher order mature fibrils and alone can convert to β-sheet rich fibrils. The multidimensional NMR study using helix-rich intermediate revealed the involvement of N-terminal and central hydrophobic segment for helical intermediate formation. The helix-rich oligomeric state also catalyzes the aggregation and amyloid formation of soluble α-Syn, suggesting seeding capacity of this intermediate similar to amyloid fibrils. Our data therefore showed the complex aggregation mechanism with existence of novel intermediate in the aggregation pathway of α-Syn, which might have significant value for drug design against PD.

## Results

### Observation of α-helix-rich intermediates during synuclein aggregation

**Figure 1:**
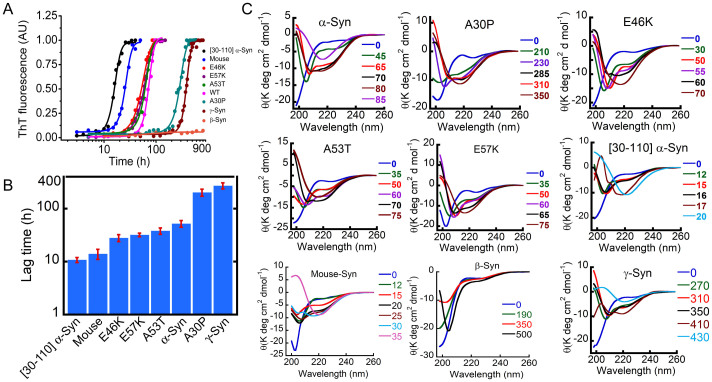
Synucleins fibrillation through helix-rich intermediate. (A) Aggregation of synucleins was monitored by ThT fluorescence. All synucleins (LMW) were incubated at 37°C with slight agitation at a concentration of 300 μM in 20 mM Gly-NaOH buffer, 0.01% sodium azide, pH 7.4. ThT fluorescence was performed at regular intervals. ThT fluorescence intensities at 480 nm were ploted against incubation time for each protein. (B) Bar diagram representation of lag time for the synucleins aggregation kinetics monitored by ThT fluorescence. (C) CD spectroscopy reveals synucleins were initially random coil and converted to β-sheet conformation via α-helix-rich intermediate. The different colored numbers indicate the incubation times (hrs). Selected CD spectra were shown.

α-Syn aggregation is observed to follow classical nucleation-dependent polymerization^52^ when its aggregation kinetics is measured by ThT (thioflavin-T) binding assay. ThT is a widely used fluorescent dye that produces strong fluorescence at 480 nm upon binding to amyloid fibrils when excited at 450 nm^53^, but does not bind to monomeric protein. α-Syn aggregation process exhibits three distinct phases with a lag phase, a subsequent rapid growth phase (elongation phase), followed by a stationary phase. In order to structurally characterize the pathway of α-Syn aggregation, CD (circular dichroism) and ThT binding was performed simultaneously during aggregation for nine different synucleins including WT (wild-type) and three familial disease-associated mutants (A30P, A53T and E46K) as listed in Supplementary Figure 1. For this study, 300 μM low molecular weight (LMW)^54^ form of all proteins were incubated at 37°C for monitoring the aggregation kinetics. Previous studies have shown that this LMW preparation mostly contains monomers along with some amount of low-order multimers of α-Syn^54^,^55^. The buffer conditions were 20 mM Gly-NaOH, pH 7.4, 0.01% sodium azide. Following Figure 1A, the ThT binding data show insignificant ThT fluorescence immediately after preparation, and during the initial period of incubation, suggesting the lack of β-sheet rich structures during the lag phase. After the lag phase, time-dependent increase in the ThT fluorescence intensity (elongation or growth phase) was observed for all synucleins (except for β-Syn) till they reach the saturation level, i.e. the stationary phase. From these sigmoidal curves, the lag times were calculated (Figure 1B) using a previously published method^56^. This analysis showed that the lag time of [30–110]α-Syn and mouse-synuclein was significantly shorter (10–12 hr) than WT (~55 hr). Compared to WT, the two disease variants A53T (lag time ~40 hr) and E46K (lag time ~30 hr) also aggregated faster. We also included the cytotoxic oligomer-prone E57K variant^54^, which also showed shorter lag time (~35 hr) compared to WT α-Syn under the conditions studied. In contrast, the A30P mutant showed slower aggregation kinetics (lag time ~210 hr) compared to WT (Figure 1A–1B). The kinetics of aggregation by γ-Syn was significantly slower (lag time ~270 hr, Figure 1B) than the other synuclein variants under study and β-Syn showed lack of fibrillation as evident from ThT binding studies (Fig. 1A)^19^. The accompanied CD data (Figure 1C and Supplementary Figure 2) suggest that at the beginning of the aggregation kinetics all synuclein variants were mostly in random coil (RC) conformation. Continuous monitoring of the CD spectra revealed that major conformational changes occurred for all these proteins (except β-Syn). β-Syn remained in mostly random coil conformation even after 500 hr of incubation with a minor conformational change. All these proteins showed aggregation process via α-helix-rich conformation (exemplified with two distinct minima: one at ~205–208 nm and the other at ~222 nm) before converting into a β-sheet-rich structure with single minima at ~218–220 nm. A53T and E46K, which are faster aggregating variants (Figure 1A), showed the appearance of a helix-rich intermediate at 35 hr and 30 hr, respectively, while for WT it was ~45 hr (Figure 1C and Supplementary Figure 2). The slow aggregation variant A30P showed a delayed appearance of the helical intermediate (~210 hr) (Figure 1C). The appearance of helical intermediate for mouse synuclein was very fast (~15 hr) compared to WT; whereas for γ-Syn it was very slow (~270 hr). The CD study of the α-Syn fibril core ([30–110]α-Syn) showed appearance of this intermediate at 12 hr (Figure 1C and Supplementary Figure 2). Interestingly, the simultaneous measurement of CD and ThT data showed that the helix-rich intermediate appeared during the fibrils growth for all the synucleins aggregation (except β-Syn) (Supplementary Figure 3). Further the conformational transition from RC to β-sheet structure of fibril does not possess a single dichroic point suggesting that the conformation transition for β-sheet rich fibril formation by α-Syn is not a simple two state process.

It is worth mentioning here that the helix-rich state also appeared when the aggregation kinetics of α-Syn was performed in PBS, pH 7.4. However, in PBS, set-to-set variation for the appearance of helix-rich intermediate was observed (Supplementary Figure 4). Moreover, the duration of helix-rich intermediate was also not consistent across the different sets. Further, the duration of helical intermediate in Gly-NaOH buffer was longer compared to PBS, which allowed us to capture and characterize the helical intermediate in more details.

The deconvolution of the CD spectra of all synuclein variants using CDPro software^57^,^58^ was consistent with the qualitative visual observation of helical intermediates in synuclein aggregation. The deconvolution of the CD spectra of initial LMW synuclein showed mostly a RC structure (>70%) with insignificant amounts of other secondary structures (Table 1 of the Supplementary Information, Supplementary Figure 5) for all the variants under study. In contrast, the deconvolution of spectra possessing the highest helix-rich conformation showed ~38% of helix, ~25% RC and ~15–20% β-sheet for most of the proteins (Supplementary Figure 5). Only [30–110]α-Syn showed a smaller content of helix (~21%) in the helix-rich intermediate state. After conversion to β-sheet, the deconvolution of the CD spectra showed ~5% helical content, ~39% β-sheet, and 30% RC content for most of the proteins with a high β-sheet content as expected (Supplementary Figure 5). Further the deconvolution of CD spectra during different time points of aggregation suggests a gradual increase in α-helix content, which reaches maxima consistent with the visual observation. After this point, α-helix content decrease drastically due to the formation of β-sheet rich fibrils (Supplementary Figure 6).

**Figure 2:**
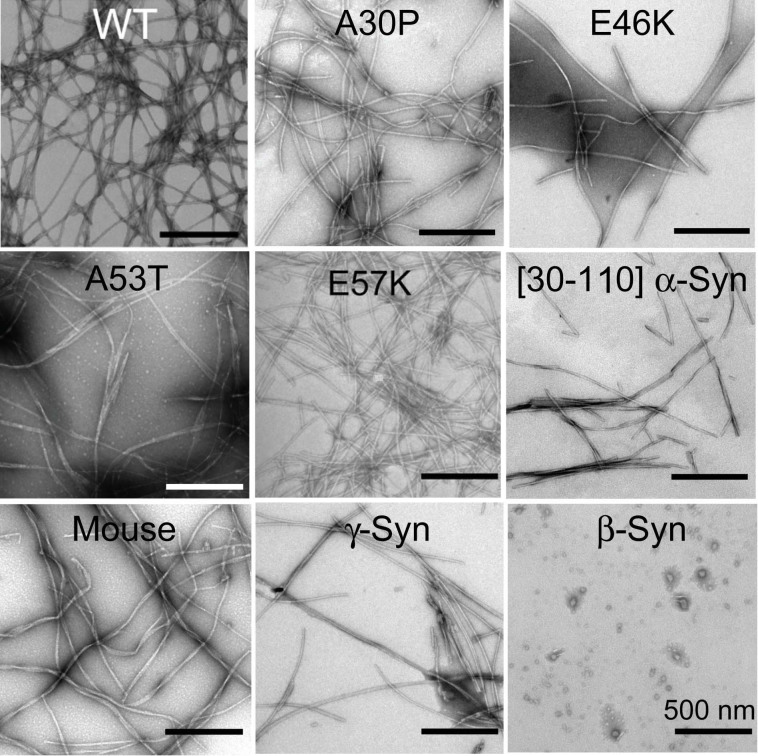
Electron microscope images of WT α-Syn and its variants. EM images of WT α-Syn, its disease associated mutants (A30P, E46K and A53T), E57K, Core, Mouse-Syn and γ-Syn showed amyloid like fibrils morphology at the end of assembly reaction. However β-Syn did not show any fibrils morphology. It formed small globular oligomers. Scale bar represents 500 nm.

To study the morphology of the aggregates at the end of the assembly reaction, electron microscopy was performed (Figure 2). All synucleins formed amyloid-like fibrils that typically displayed a helical ultra-structure and varying degrees of lateral association except β-Syn. β-Syn formed small, low order globular oligomers. Close inspection of the fibrils suggested that fibrils were largely composed of multiple individual filaments (diameter ~3–4 nm), resulting in overall diameters ranging from 15 to 25 nm.

Collectively, the ThT fluorescence and CD data measured during the entire period of synuclein aggregation suggest that in the lag phase, all the synucleins displayed mostly a random coil-like structure, that the helix-rich conformations appeared at the onset of fibril growth and persisted till the mid-elongation phases of the aggregation kinetics followed by a β-sheet conformation at the end (Figure 1 and Supplementary Figure 2–3).

In this context, it is important to mention that helix initiation in conformational transition during α-Syn aggregation is defined based on the first observation of two minima in CD spectra; one at ~205–208 nm and another at ~222 nm. Duration of helix is defined by the time between the helix initiation and final helix observed (before the first β-sheet appearance with CD spectra of single minima at ~218–220 nm). However, the onset of helix conformation and duration of helix is solely determined by visual inspection and are qualitative. Since we performed CD spectroscopy at regular time intervals (not in a continuous manner) the actual time for these conformational transitions might vary from the visual observation.

### Duration of helix-rich intermediates is dependent on the aggregation propensity of α-Syn

**Figure 3:**
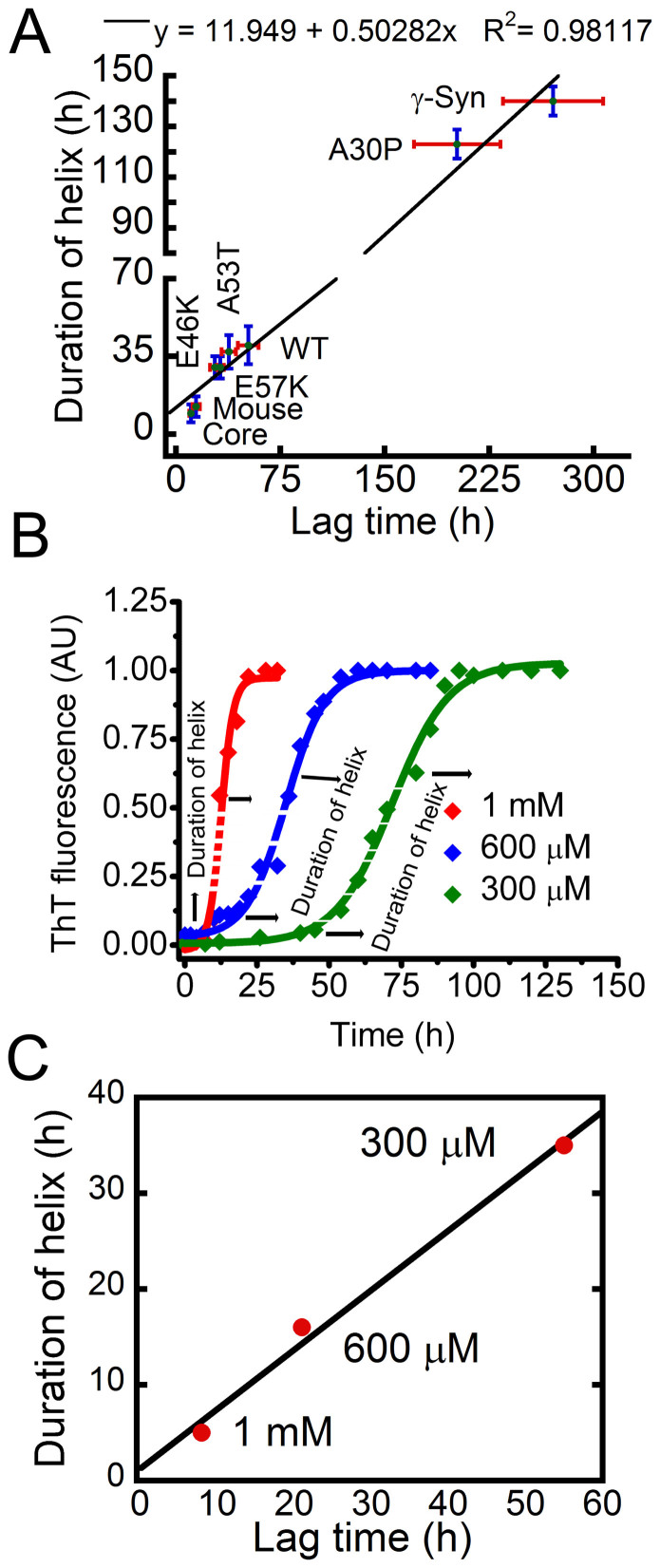
Duration of helix-rich intermediates is dependent on the aggregation propensity of α-Syn. (A) Duration of helix and lag time for different synucleins follows a linear correlation. (B) Concentration dependent aggregation kinetics of WT α-Syn measured by ThT binding assay. (C) Corelation plot for the lag time and duration of helix for WT α-Syn at different concentration (1 mM, 600 μM and 300 μM). Duration of helical intermediate is lowest for 1 mM and longest for 300 μM concentration of α-Syn.

The correlation plot (Figure 3A) of lag time (extracted from ThT binding data) versus duration of helical intermediates (observed by CD) of the various synucleins suggest that synucleins with shorter lag time showed a shorter duration of helical intermediates compared to synucleins with a longer lag time. Supplementary Figure 7A and 7B also showed the linear correlation between lag time and appearance of helix/saturation time of the individual mutants. To rule out the possibility that the duration of helical intermediates is not specific to individual synuclein variants, but rather depend on the relative tendency of aggregation, the aggregation of WT α-Syn was studied with two other different concentrations (600 μM and 1 mM) in 20 mM Gly-NaOH buffer, pH 7.4, 0.01% sodium azide. The aggregation kinetics was studied simultaneously by ThT binding (Fig. 3B) and CD (Supplementary Figure 8) spectroscopy. The lag time for 1 mM, 600 μM and 300 μM of α-Syn were of ~8 hr, ~21 hr and ~55 hr, respectively (Figure 3B–3C). Moreover, for all these cases, the helical intermediates appeared at the beginning of the elongation phase (Figure 3B). The correlation plot of the lag time versus duration of helix appearance (Figure 3C) also suggest that under slow aggregation conditions (i.e. 300 μM concentration), α-Syn shows the longest period of helical intermediate, which shortens upon increase in protein concentration. The data indicates that under faster aggregating conditions, both RC → helix and helix → β-sheet transitions are faster resulting in a short duration of helical intermediate appearance.

### Helix-rich intermediate of α-Syn identified by FTIR

**Figure 4:**
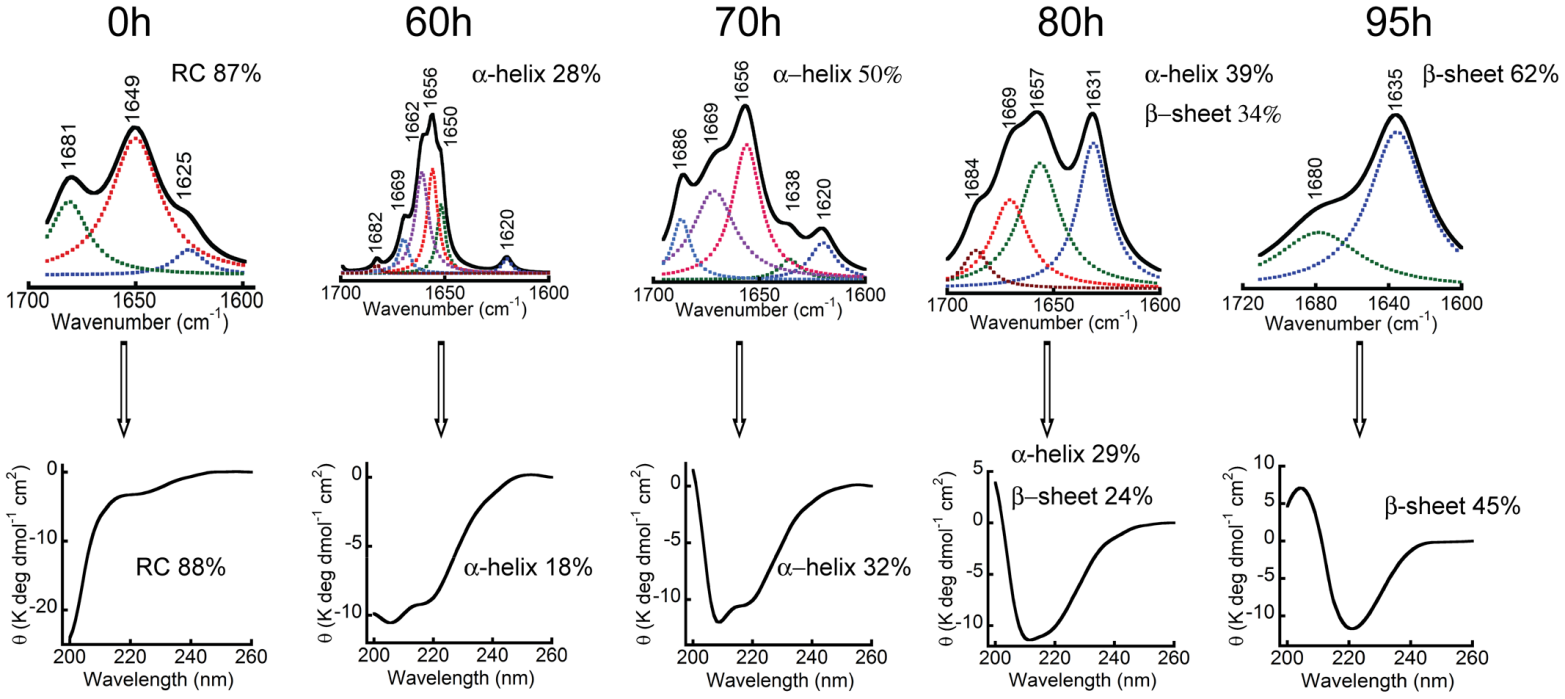
Secondary structural changes monitored by FTIR spectroscopy. Curve fitted FTIR spectra in the region corresponding to amide-I band showing the different secondary structural content of α-Syn species at different stages of aggregation (Upper panel). FTIR spectral data suggest predominate random coil structure at the beginning that eventually converted to β-sheet rich structure via α-helical intermediate during α-Syn aggregation. Corresponding CD spectra of the identical samples were shown in lower panel.

To further confirm that the aggregation of α-Syn involves a helix-rich intermediate formation, WT α-Syn aggregation was monitored by FTIR and CD spectroscopy. The LMW α-Syn at a concentration of 300 μM was incubated at 37°C with slight agitation (~50 r.p.m). Simultaneously, FTIR and CD spectroscopy was performed at regular intervals. The FTIR spectra in the range of 1600 cm^−1^ to 1700 cm^−1^ (amide-I band) were analyzed to determine the protein secondary structure^59^. After immediate dissolution and preparation (time point 0), FTIR spectra of soluble α-Syn showed a major absorbance peak at 1649 cm^−1^, indicating the presence of mostly random coil-like structure. The deconvolution of FTIR spectra at this point showed ~87% of random coil structure, consistent with CD spectra (88% random coil by CD deconvolution using CDPro software^57^ (Figure 4). With the progression of time when the incubated solution showed helical conformation in CD, FTIR spectra were measured. At 60 h, the intensity of the absorbance peak at 1649 cm^−1^ decreased and showed several other FTIR absorption peaks with a major absorption peak appeared at 1656 cm^−1^ indicating the formation of a helix-rich intermediate. This absorption peak at 1656 cm^−1^ was most prominent at 70 h. The deconvolution of FTIR spectra at 60 h and 70 h showed ~28% and 50% helicity, respectively. The deconvolution of CD spectra by CDpro^57^ showed 18% and 32% helix at 60 h and 70 h, respectively. On further incubation, another major peak appeared at 1630 cm^−1^ (as shown for 80 h) and intensity of the peak at 1656 and 1630 cm^−1^ became almost equal suggesting a significant population of both helix-rich intermediate and β-sheet-rich structure in the mixture (~39% helix and ~34% β-sheet content). The deconvolution of corresponding CD spectra at 80 h also revealed almost equal amount of helix (~28.6%) and β-sheet (~25.3%) content. The subsequent incubation at time point 95 h, for which the CD spectra of the solution predominately showed β-sheet secondary structure (Fig 4)), the FTIR study of the solution showed major absorption peaks at 1635 cm^−1^ corresponding to β-sheet-rich amyloid fibrils (~62% β-sheet content). Therefore, the FTIR data confirmed the findings of the CD data thereby strengthening the notion that a helix-rich intermediate is formed during α-Syn fibrillation.

### Temporal change in secondary structure and morphology during α-Syn aggregation

**Figure 5:**
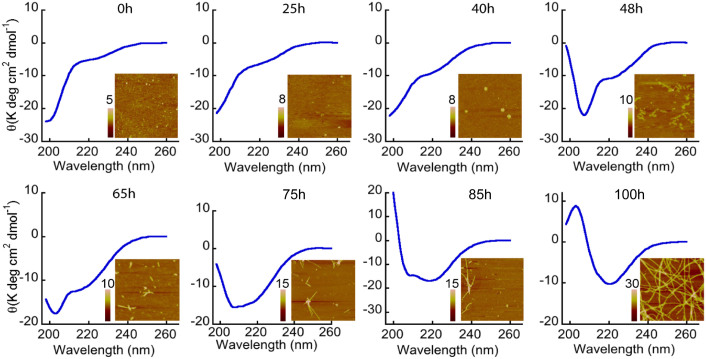
Temporal changes in secondary structures and morphology of α-Syn during amyloid formation. CD and AFM study of α-Syn during aggregation showing a transition from RC to β-sheet via a helix-rich intermediate. AFM study showing small oligomers/amorphous structures at the beginning, the helix-rich state appears to be composed of a heterogeneous population of different species including spherical oligomers and small filaments before assembled to mature fibrils (at 100 hr). AFM images are squares of 2.5 μm. Heights scales are depicted in individual AFM images.

To study and correlate the temporal changes in secondary structure with morphology during α-Syn aggregation, AFM and CD were performed simultaneously at regular intervals during WT α-Syn aggregation in 20 mM Gly-NaOH buffer, pH 7.4, 0.01% sodium azide at 37 °C. In line with other experiments discussed, immediately after preparation, WT α-Syn was unstructured based on CD analysis, and the AFM showed mostly low order oligomers/amorphous structure at 0 hr (Figure 5). With the progression of the assembly, WT α-Syn showed gradual transformation into more ordered structure as θ_222_ became more negative during aggregation. Similarly, AFM showed a gradual increase in oligomers size during incubation. At 48 hr, WT α-Syn transformed into a partial helix-rich structure with two distinct minima at ~205 and ~222 nm, respectively, with small and twisted protofibrils observed in AFM. The continuous CD studies further revealed a gradual increase in the helical content in the CD spectra of α-Syn. At the time of maximal population of the helix-rich intermediate (at 75–85 hr), α-Syn exhibited a heterogeneous mixture of oligomers, protofibrils and short filaments in the AFM measurements (Figure 5). The average heights of oligomers were of 5–8 nm. At the end of the assembly reaction, α-Syn converted to mature and thick β-sheet-rich amyloid fibrils (15–30 nm in height) as expected (Figure 5).

### Sequence segments of α-Syn critical for the formation of helical intermediates

**Figure 6:**
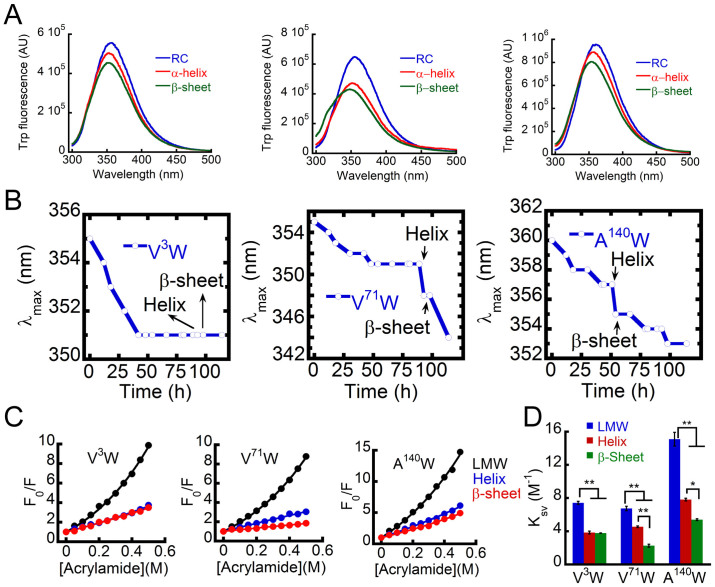
Sequence segments of α-Syn critical for the formation of helical intermediates probed by Trp fluorescence study. (A) Trp emission spectra showing gradual blue shift of λ_max_ of for V^3^W (left), V^71^W (middle) and A^140^W (right) during RC → α-helix → β-sheet transition. (B) Changes of Trp fluorescence intensity maxima (λ_max_) during helix-rich intermediate and amyloid formation by Trp-substituted WT α-Syn. The large λ_max_ change was evident for V^71^W and A^140^W during helix → β-sheet transition. (C) Modified Stern-Volmer plots of Trp quenching for [V^3^W]α-Syn, [V^71^W]α-Syn and [A^140^W]α-Syn using acrylamide. Quenching of site-specific Trp mutants at three different stages of incubation were carried out (freshly prepared low molecular weight (LMW), helix-rich intermediate and at the time of first β-sheet appearance (β-sheet)). F indicates maximum Trp fluorescence intensity in presence of different acrylamide concentrations and F_0_ stands for maximum Trp fluorescence intensity in absence of acrylamide. (D) Stern-Volmer quenching constants of site-specific Trp variants of freshly prepared LMW, helix-rich intermediate and immediately after conversion to β-sheet.

To understand the involvement of the N-terminal, C-terminal and the NAC regions in the helical intermediate formation and subsequent β-sheet formation, three Trp substituted WT α-Syn (V^3^W, V^71^W and A^140^W) were studied. Similar to WT α-Syn, the Trp substituted proteins also showed RC → helix → β-sheet structural transition and formed amyloid fibrils (Supplementary Figure 9A–B). We determined the microenvironment and solvent exposure of Trp by measuring λ_max_ shifting and acrylamide quenching of fluorescence, respectively at different stages of aggregation for these Trp substituted WT α-Syn. Simultaneous measurement of CD and Trp fluorescence showed the gradual blue shifting of λ_max_ of Trp fluorescence during the helical intermediate formation in all the proteins (Figure 6A–B). From the data it is clear that although a noticeable blue shift of λ_max_ occurred during the helix → β-sheet transition for V^71^W and A^140^W, λ_max_ remained almost constant during helix to β-sheet transition for V^3^W (Figure 6A–B). The blue shifting of λ_max_ for V^71^W was 3 nm, whereas for A^140^W was 2 nm on the helix to β-sheet transition. When λ_max_ was determined by a second order polynomial curve fitting rather than direct inspection, we also find a similar trend for the blue shift of λ_max_ (Supplementary Figure 10). However, the absolute value for the blue shift of λ_max_ was slightly differed from visual observation. The blue shift of λ_max_ for V^71^W was of 2.6 nm (obtained from curve fitting) instead of 3 nm (obtained from visual inspection) during helix → β-sheet transition; whereas for A^140^W, the curve fitting resulted 2.1 nm blue shift, which is almost identical (2 nm) with visual observation. The data thus suggests changes in the microenvironment of all the three Trp positions during random coil to helix transition; whereas upon helix to β-sheet transition, changes in the microenvironment occur only for V^71^W and A^140^W. The microenvironment of V^3^W remained essentially unchanged upon helix to β-sheet transition.

To study the Trp solvent exposure, acrylamide-quenching studies were performed (Figure 6C). The quenching data showed that the helical and β-sheet-rich species were more solvent protected compared to their corresponding monomeric state of α-Syn. The data were fitted with the modified Stern-Volmer equation^60^ and the Stern-Volmer quenching constant (K_sv_) was determined (Figure 6D). The data for V^3^W indicate that the burial of the N-terminus occurred during conversion from random coil (K_sv_ 7.4 M^−1^) to α-helix (K_sv_ 3.8 M^−1^), which does not further change during conversion to the β-sheet (K_sv_ 3.7 M^−1^), consistent with the λ_max_ shift data. However, the V^71^W and A^140^W showed a significant lowering of the Stern-Volmer quenching constant both from random coil to helix and subsequently to the β-sheet state (Figure 6D). The data further suggests that in the helical intermediate, A^140^W is more solvent exposed (K_sv_ 7.8 M^−1^) compared to V^71^W (K_sv_ 4.5 M^−1^) and V^3^W (K_sv_ 3.8 M^−1^). Further, on the conversion from the helix to β-sheet-rich structure, K_sv_ for V^71^W becomes almost half. While for A^140^W, the K_sv_ changes from 7.8 M^−1^ at helix-rich state to 5.4 M^−1^ at β-sheet state. The data therefore indicates that gradual burial of central, N- and C-terminal regions accompanied the random coil to helix conversion, whereas significant conformational transition occurred during the helix to β-sheet conversion in the central region and C-terminal segments of α-Syn, respectively.

### Segments responsible for helix formation determined by NMR

**Figure 7:**
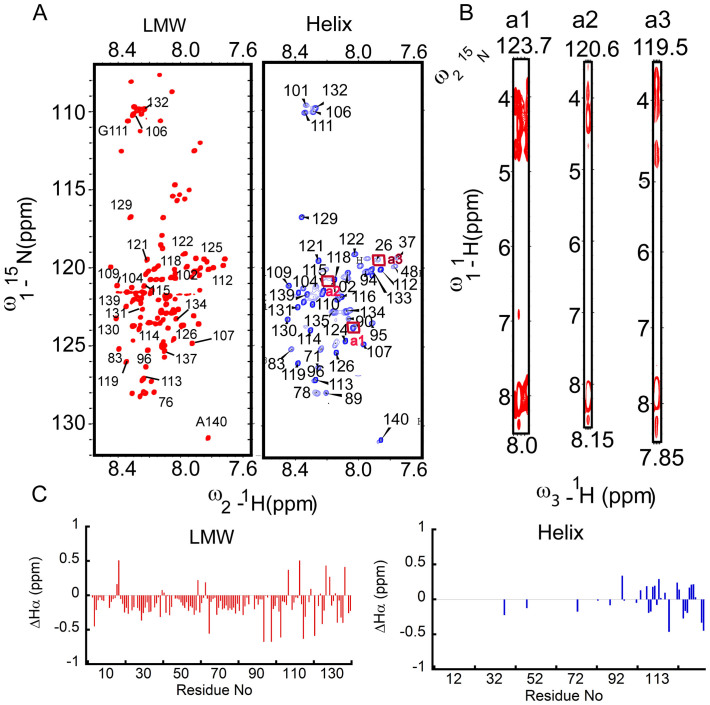
Segments responsible for helix formation determined by NMR. (A) 2D heteronuclear single quantum coherence (HSQC) NMR spectroscopy of LMW (left panel), helix-rich state (right panel). (B) NOESY-HSQC experiment of helix-rich intermediate H^N^-H^N^ connections originating from the low-intensity peaks. (C) H^α^ secondary chemical shifts of resonances for LMW (left panel) and helix-rich state (right panel).

To further confirm the formation of helical intermediates during synuclein fibrillation and in order to further corroborate the segment responsible for helix formation, 2D heteronuclear single quantum coherence (HSQC) NMR spectroscopy was performed. ^15^N-labeled α-Syn was prepared as described in method section and used for acquiring NMR spectra at 15°C. Figure 7A (Left panel) shows well resolved [^15^N, 1^H^]-HSQC spectra of LMW α-Syn with very sharp peaks and small chemical shift dispersion along the proton dimension, indicating an intrinsically disordered protein species as expected^61^. Next, the LMW ^15^N-labelled α-Syn sample was incubated at 37°C for fibrillation in parallel to CD studies. When helical intermediates were detected in the CD study (~65 h), samples were taken for the NMR measurements. In this state, numbers of visually observed peaks were found to be less compared to LMW α-Syn (Figure 7A, right panel). We also noticed that these peaks that appeared in the helix-rich states were almost overlapping with the NMR peaks of LMW state.

We used 3D TOCSY-HSQC together with assignment transfer to assign the NMR spectra of helix-rich state. It was found that the sharp peaks in the helix-rich state were mostly originated from the C-terminal amino acid residues (amino acid 100–140). These results indicated the involvement of the N-terminus and hydrophobic central region in helix formation, whereas, C-terminus remained flexible. Importantly to mention, presence of structured N-terminus in α-Syn oligomers has been also reported in many studies^62^. Further, our NOESY-HSQC experiment showed few H^N^-H^N^ connections originating from the low-intensity peaks (Figure 7B), substantiating the possibility of helical conformation adopted by the N-terminus and/or the central region of α-Syn. We further calculated the Hα secondary chemical shifts obtained from the helix-rich samples and compared with the LMW H^α^ secondary chemical shifts. In the helix-rich state, the C-terminal residues populate their ϕ and ψ in the broad β-region while the N-terminus residues adopt ϕ and ψ corresponding to helical conformation (Figure 7C). Although our current NMR spectroscopic data is limited to the qualitative information about the helix-rich state, the details structural characterization of this intermediate may require further study with stabilized intermediate and use of solid-state NMR spectroscopy.

### Isolation and characterization of the helical intermediate

**Figure 8:**
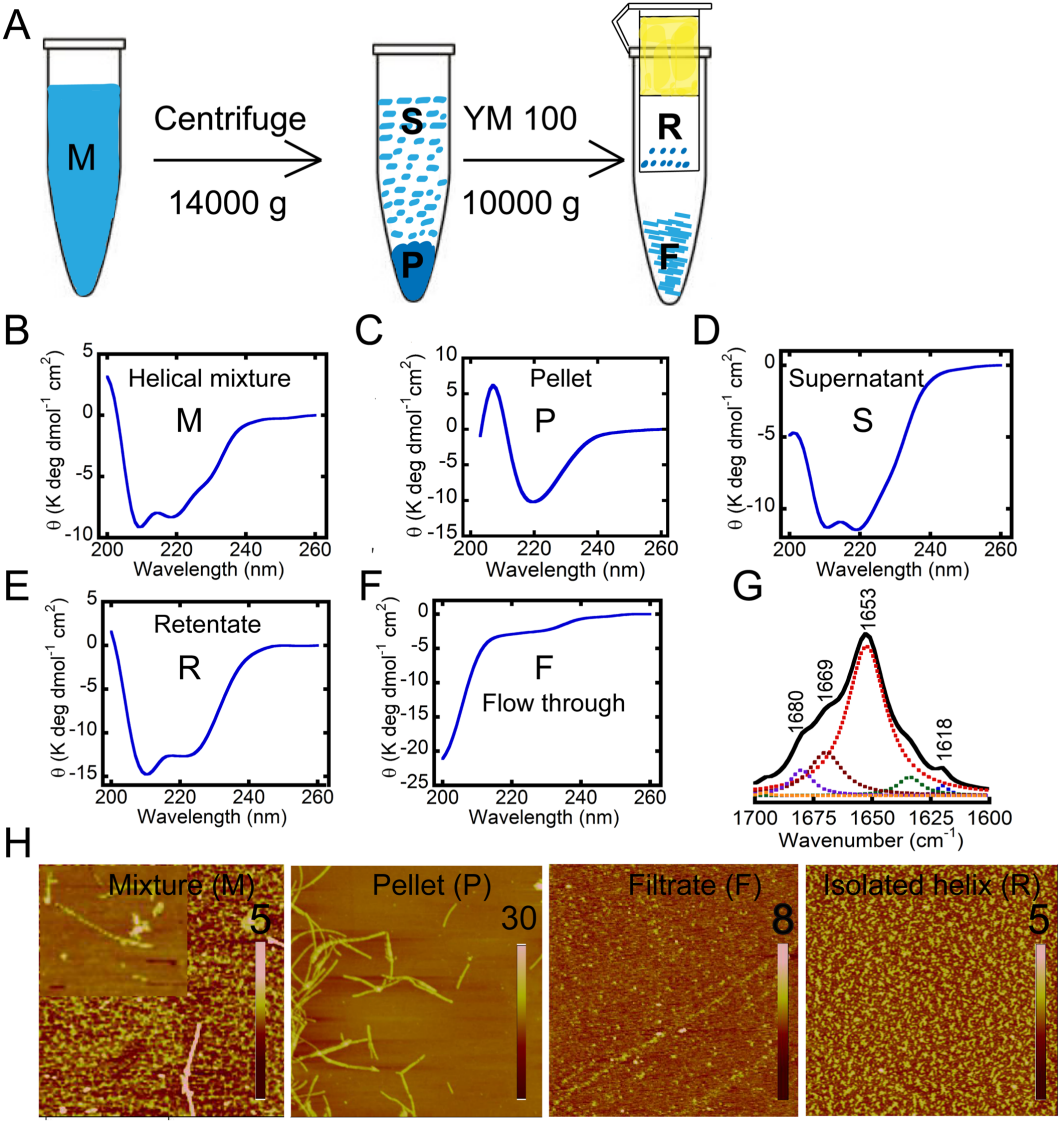
Isolation and biophysical characterization of helical intermediate. (A) Schematic showing the isolation of helical intermediate. Secondary structure determined by CD spectroscopy for (B) helical mixture, (C) pellet, (D) supernatant, (E) retentate (isolated helix) and (F) flow through containing soluble α-Syn. (G) FTIR spectroscopy of isolated helix showing most intensity peak at 1653 cm^−1^ corresponding to helix conformation. (H) Morphological analysis for various isolated α-Syn species. AFM images are square of 5 micron and height scales are shown in individual AFM images.

The question arises whether the helical intermediate that appeared on the onset of rapid growth phase is the mixture of different α-Syn species (different aggregates with different secondary structure) or a single structural/oligomeric entity. The latter scenario was hypothesized for the helix-rich intermediate in IAPP fibrillation^46^,^63^. However, our AFM study showed that different aggregates species are present in the helix-rich state along with a few matured fibrils. If the intermediate is a mixture of different α-Syn species, then it might be possible to isolate it from the other α-Syn species. Following this hypothesis, 300 μM WT α-Syn was incubated for aggregation at 37 °C. When the helical intermediate appeared as probed by CD spectroscopy (Figure 8B), the solution was centrifuged (Figure 8A) at 14000 g for 15 minutes at 4°C in order to remove the supernatant from the fibrillar aggregates (in the pellet (P)). The resuspended pellet fraction in solution showed predominately a β-sheet conformation in the CD spectrum as expected (Figure 8C), while the soluble supernatant (S) fraction showed a helix-typical CD spectrum (Figure 8D). When the supernatant fraction was further allowed to pass through a 100 kDa MWCO cut off filter, the retentate (R) fractions showed a prominent helix-typical CD spectrum, whereas the filtrate/flow through (F) showed a random coil-like spectrum (Figure 8E–8F). The present data therefore suggest that the helical intermediate appearing during synucleins aggregation is a distinct oligomeric entity with a molecular weight more than 100 kDa. The concentration measurement of helix-rich state and soluble α-Syn suggests that in the helix-rich state, the isolated helical α-Syn was 50%, soluble α-Syn was 25% and rest 25% was preformed fibrils.

The FTIR spectrum of the isolated helix species (i.e. fraction R) strengthens the present finding of a distinct helical oligomeric species by showing a major absorption peak at 1653 cm^−1^, characteristic of a helical conformation (Figure 8G). However, other less intense peaks ~1669 cm^−1^, ~1620 cm-1 and 1680 cm^−1^ that correspond to turn and cross β-sheet structures are also identified, suggesting that either the helix-rich intermediate may also possess subtle degree of other secondary structural elements or the present purification protocol for the helical species is unable to completely get rid of all the other type of aggregates. The different species isolated were further morphologically characterized by AFM. The pellet (P) fraction showed mostly fibrillar morphology of ~15 nm in height, while the filtrate (F) showed lower order assemblies of height ~3/4 nm. The isolated helix-rich species (fraction R) showed oligomers and thin protofilaments-like structure of height ~5 nm (Figure 8H).

### Fibril formation by isolated helix-rich intermediate

**Figure 9:**
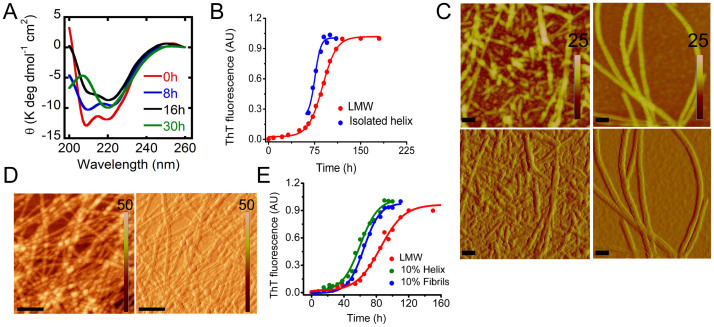
‘On pathway' α-helical intermediate during synuclein fibrilization. (A) CD spectra during aggregation showing conversion from isolated helix-rich state to β-sheet upon incubation. (B) Kinetics of aggregation of LMW α-Syn and isolated helix monitored by ThT. (C) Morphological analysis of fibrils formed from isolated helix (left panel) and LMW α-Syn (right panel) by AFM. Scale bar are 500 nm. Height scales (upper panel) and amplitude scales (lower panel) are shown in individual AFM images. (D) Morphological characterization of the fibrils formed by the isolated helix in presence of externally added fibrils by AFM showing regular characteristic of thin and long fibrils morphology. Scale bar are 500 nm. Height scales (left panel) and amplitude scales (right panel) are shown in individual AFM images. (E) Seeding capacity of isolated helical intermediate and fibrils. The presence of 10% (v/v) helical intermediate and fibrils in a sample containing 300 μM LMW α-Syn reduced the lag phase of α-Syn fibril formation. α-Syn without any seeds was used as a control.

To study whether isolated helix-rich intermediate alone can be matured to β-sheet rich fibrils, we isolated the helix-rich state and incubated at 37°C for fibrillation. The conformational transition and amyloid formation was monitored using CD and ThT fluorescence, respectively. The CD data suggest that helix-rich state alone can get converted to β-sheet rich structure upon incubation (Figure 9A). The ThT fluorescence study with isolated helix-rich intermediate during incubation suggests that this intermediate matured to fibrils without any lag phase in contrast to α-Syn aggregation observed from soluble, unstructured α-Syn (Figure 9B). The AFM study of the fibrils formed from isolated helix showed mostly short filaments in contrast to the long fibrils that are formed by soluble LMW α-Syn (Figure 9C). The short filaments, which were formed from the helix rich intermediate, did not further convert to long fibrils even after long incubation (data not shown). However, the fibrils formed from isolated helix showed slightly higher amount of ANS binding (Supplementary Figure 11A) and almost similar toxicity (Supplementary Figure 11B–C) as that of the fibrils formed by soluble LMW α-Syn. We suggest that the presence of fibrils is required for helix-rich state to mature to long fibrils. To study this, we incubated isolated helix-rich intermediate in the presence of 10% (v/v) fibrils and incubated for 2 days. The AFM data showed the formation of long fibrils (Figure 9D) confirming that helix-rich intermediate interacts with preformed fibrils in the early elongation phase for the maturation of fibril formation.

### The helix-rich intermediate catalyzed α-Syn fibrillation

In α-Syn aggregation, preformed amyloid fibrils is known to accelerate the aggregation kinetics of soluble α-Syn^52^. To examine whether the helix-rich intermediate can template and accelerate α-Syn aggregation, 300 μM freshly prepared LMW α-Syn in 20 mM Gly-NaOH buffer, pH 7.4, 0.01% sodium azide was incubated in presence and absence of 10% isolated helical intermediate (Figure 9E). 300 μM α-Syn in presence of 10% preformed fibrils was also studied as a positive control. Important to note that, in contrast to traditional seeding experiments, where fibrils were sonicated into broken fibrils, we used unsonicated helical intermediate because the sonication may affect its conformation. To keep appropriate control, we did not perform sonication of fibrils as well. It was found that in the presence of the isolated helix-rich intermediate, the lag time of aggregation was significantly reduced (Figure 9E). The lag time of freshly prepared LMW α-Syn in the absence of any seeds was ~55 hr, while the lag times were significantly reduced to ~43 hr and ~38 hr in the presence of 10% preformed fibrils and isolated helix-rich intermediate, respectively. It was shown recently that aggregation kinetics in the presence of seeds (broken fibrils), which are more compatible as nucleus for aggregation and work through the bulk of the solution^64^. In contrast, the unsonicated fibrils were found to be surface active and retained adsorbed at air-water interface^64^ and accelerates the aggregation through the surface (surface catalyzed reaction) of the solution^64^. Therefore, the observed acceleration of α-Syn aggregation kinetics might be due to the effect of surface of the species for templating the aggregation.

### Hydrophobic surface exposure and toxicity of helical intermediates

**Figure 10:**
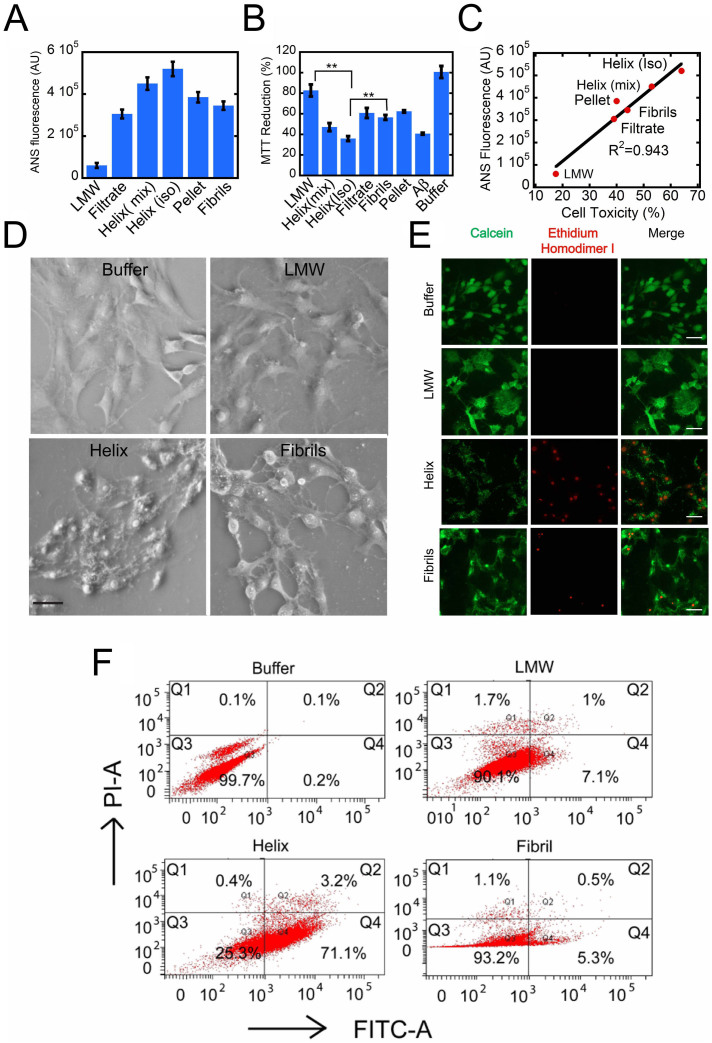
Hydrophobic surface exposure and toxicity of helix-rich intermediate. (A) ANS fluorescence of different α-Syn species indicate maximum exposed hydrophobic surface for isolated helix-rich state. (B) MTT reduction by SH-SY5Y cells in presence of various α-Syn species showing higher cytotoxicity (less MTT reduction) of isolated helix and material comprising helix-rich intermediates. LMW, matured fibrils (5 days aged sample) and Aβ (25–35) fibrils were used as controls. (C) Correlation plot of ANS binding (hydrophobic surface exposure) and MTT reduction-based cytotoxicity indicates that species possessing larger hydrophobic exposed surfaces are more toxic. (D) Phase contrast images for SHSY-5Y cells treated with different species of α-Syn indicating maximum cell death for isolated helix followed by fibrils. However the morphology of cells treated with LMW remained in normal state similar to control cells (treated with buffer). Scale bar represents 100 micron. (E) The calcein and EthD-1 staining showing defective morphology with damages of cell body/neuritis of cell treated with helix-rich intermediate. Cells treated with either LMW or buffer control showed no significant of cell death/defective cell morphology. Scale bar represents 200 micron. (F) The flow cytometry analysis showing that induction of early apoptosis by cells treated with helix-rich state. The cells treated with matured fibrils and LMW samples did not show significant toxicity. Buffer was used as a control. Quadrants Q1, Q2, Q3 and Q4 represent dead cells, late apoptotic/necrotic cells, live cells and early apoptotic cells, respectively.

It has been suggested that the extent of hydrophobic surface exposure may play a significant role in cellular toxicity of protein aggregates^65^,^66^. To determine the hydrophobic surface exposure of the helical intermediate and other α-Syn species, 1-anilinonaphthalene-8-sulfonate (ANS) binding study was performed with different species of α-Syn. ANS is a traditional dye frequently used for protein folding studies to detect a molten globule state^67^. It binds to exposed hydrophobic surfaces of proteins and, therefore, is able to monitor the relative hydrophobic surface exposure during folding and aggregation. To do the ANS binding, different species of α-Syn were isolated as described in the method section. Freshly prepared LMW was used as a negative control. The helix-rich state showed maximum ANS binding followed by isolated fibrils from the pellet purification step and fibrils collected at the end of the aggregation kinetics (Figure 10A and Supplementary Figure 12). LMW α-Syn showed least ANS binding. The increased hydrophobicity of the helix intermediate must come either from the oligomer formation or/and a structural rearrangement that brings together in closely space hydrophobic amino acid side chains. The data suggest the isolated helical intermediate contained more exposed hydrophobic surface area compared to fibrils and exposed hydrophobic surface area is least for the soluble protein (Figure 10A Supplementary Figure 12).

Further, to study the toxicity of the helix-rich intermediate, a cell-based toxicity assay (MTT 3-(4,5-Dimethylthiazol-2-yl)-2,5-diphenyltetrazolium bromide)^68^) using SH-SY5Y neuroblastoma cell line was performed (Figure 10B). The isolated helical intermediate along with the pellet fraction (P), the filtrate (F) (see Figure 8A), and non-isolated sample that showed a helix-rich signal in the CD spectrum (termed helix-rich mixture in the following section) were used for the toxicity study (Figure 10B). Freshly prepared LMW α-Syn, α-Syn fibrils (5 days old), and Aβ fibrils were used as controls. The MTT reduction data showed that isolated helix (R) showed lowest MTT reduction ~35% followed by helix mix ~47% and fibrils ~55%. However, pellet (P) and fibrils showed almost similar MTT reduction. The LMW α-Syn showed highest MTT reduction (~82%) as expected. The positive control of Aβ (25–35) fibrils showed ~40% MTT reduction. This suggests that the helix-rich intermediates are highly cytotoxic compared to β-sheet-rich fibrils and LMW α-Syn. The toxicity and hydrophobic exposure comparison plot of the helix-rich materials (Figure 10C) reveal that there appears to be a correlation between hydrophobic surface exposure and cytotoxicity.

Consistent with MTT data, a similar observation was also obtained when cell morphology of SH-SY5Y cells that treated with different species of α-Syn were imaged under phase contrast microscope. It was found that almost similar cell morphology was observed when cells were treated with LMW α-Syn compared to buffer control after 24 hr of incubation (Figure 10D). However, the cells treated with helix-rich mixture and fibrils showed abundant cells with distorted morphology (damaged cell body and neurites). Further, the cell death was analyzed using calcein AM and ethidium homodimer (Eth D1) staining. Calcein AM stains the live cells^69^,^70^ while Eth D1 detects the DNA of dead cells^55^,^71^. The calcein AM and Eth D1 staining reveals mostly normal status of cells when treated with LMW α-Syn (Figure 10E). However, Eth D1 staining was mostly found when cells were treated with the helical intermediate and less extent with fibrils. Consistent with our MTT assay, phase contrast imaging, calcein AM/Eth D1 staining also suggested that the isolated helix-rich state is more cytotoxic compared to the matured fibrils (Figure 10E).

Finally to quantify the toxicity, cell death in the presence of different α-Syn species were analyzed by Annexin V and propidium iodide (PI) staining of the SH-SY5Y cells using flow cytometry analysis^55^. It was known that in an early apoptosis event, Annexin V binds to the phospholipid phosphatidylserine (PS) that is translocated from the inner to the outer leaflet of plasma membrane (Annexin V+). When staining of Annexin V-FITC is performed along with the live/dead dye propidium iodide (PI), the analysis allows identification of cells undergoing late apoptosis and/or necrosis (Annexin V+, PI+). In contrast, binding of both Annexin V and PI would be negative (Annexin V**−**, PI**−**), when cells were completely viable. The dead cells, however, would bind mostly with PI (Annexin V**−**, PI+). Therefore, staining with Annexin V-FITC along with the live/dead dye propidium iodide (PI) allow differentiating whether cells are viable or undergoing early/late apoptosis and/or necrosis (Annexin V+, PI+)^72^,^73^. Importantly, as this study requires the α-Syn species with higher concentrations, helix-rich mixture (instead of isolated helix) was used. As a control, preformed fibrils and freshly prepared LMW α-Syn were also used. The data shown in Figure 10F suggests that 70% cells were undergoing early apoptosis in presence of the helix-rich intermediate (helix _mix_) while cells in presence of freshly prepared LMW α-Syn and fibrils thereof showed only ~10% and ~7% early apoptosis (Figure 10F). In the presence of buffer, most of the cells were viable. The data clearly suggest that under the experimental conditions tested, helix-rich material of WT α-Syn is cytotoxic.

Although all these different methods of toxicity clearly showed higher toxicity of helix-rich state, the possibility of transformation of this helical intermediate into higher order aggregates in cell culture environment during experimental time period cannot be ruled out. It is however quite difficult to evaluate directly the protein conformation responsible for cellular toxicity. However, the toxicity studies of the helical intermediate along with appropriate controls of freshly formed β-sheet rich fibrils and mature β-sheet fibrils indicate that the higher toxicity observed for helix-rich state could be due to the oligomeric helical intermediate rather than the higher order β-sheet rich aggregates that may form in cell culture experiment.

## Discussion

Aggregation of proteins into amyloid fibrils is a multistep process where monomeric protein gradually gets converted into various oligomeric forms before forming matured fibrils^41^,^44^,^74^,^75^,^76^. Several *in vitro* aggregation studies have shown that natively unstructured and folded proteins undergo large conformational transitions of RC → β-sheet^19^ and helix → β-sheet, respectively upon amyloid formation^77^,^78^,^79^,^80^,^81^,^82^. However, several studies have also suggested that natively unstructured proteins may undergo structural transition of RC → β-sheet through a helix-rich intermediate during amyloid formation^41^,^42^,^46^,^47^,^83^,^84^. Teplow and co-workers for the first time reported the existence of a helix-rich intermediate in the amyloid formation pathway of Aβ protein associated with AD^41^. This helix-rich intermediate was mostly oligomeric in nature and was suggested to be an on-pathway intermediate of Aβ aggregation. Subsequently, many studies showed the presence of a helical intermediate during the aggregation of IAPP^42^,^43^,^44^ (associated with type 2 Diabetes) and several other amyloidogenic proteins/peptides^48^,^49^,^50^. Raleigh and co-workers have shown that helix-rich intermediate also populate during fibril formation by Pro-IAPP in presence of glycosaminoglycans such as heparan sulfate^45^.

Although most of the conformational studies of α-Syn aggregation showed random coil → β-sheet structural conversion^19^ recent studies have shown that condition that favored partial helix structure (using organic solvents) of α-Syn facilitates amyloid formation^30^,^31^,^85^,^86^. Anderson *et al* showed α-helix rich state also populates during aggregation and fibrillation of α-Syn. However, its role in synuclein fibrillation, mode of toxicity, and exactly where it appears in the aggregation pathway is not known yet. Further biophysical and structural characteristics of this intermediate have not been studied yet. In the present study, we performed the structural and morphological characterization of the entire aggregation pathway of α-Syn *in vitro*. Our *in vitro* aggregation studies using CD and FTIR combined with ThT binding studies (Figure 1A, 1C, 4 and Supplementary Figure 2) showed that α-Syn remains in an unstructured state immediately after preparation. During aggregation, it transforms into an oligomeric, partial helix-rich state, which subsequently converts to β-sheet-rich fibrils (Supplementary Figure 13). The three familial disease-associated mutants (A30P, A53T and E46K) of α-Syn as well as E57K, γ-Syn, mouse-Syn and [30−110]α-Syn also showed RC → helix → β-sheet structural transitions during their aggregation (Figure 1C and Supplementary Figure 2). We thus report here the existence of α- helix-rich intermediate (in the absence of any organic solvent) during the aggregation of all synucleins (except β-Syn) under study. We believe that careful removal of preformed seeds, use of low ionic strength buffer (Gly-NaOH, pH 7.4) and regular monitoring of the conformational transitions (3 to 5 days monitoring and measuring CD spectra at regular intervals) enable us to observe this α-helical intermediate during synuclein aggregation pathway.

Further, our aggregation study revealed that all synucleins form helix-rich intermediate at the beginning of the elongation phase of aggregation (Supplementary Figure 3) that extended up to mid-elongation phase after which all proteins converted to β-sheet structure.

Our AFM morphological characterization study during aggregation revealed that this helix-rich state is a mixture of oligomeric species of various sizes as well as small fibrillar aggregates. Further, the residue level structural characterization using NMR suggest that first 100 residues containing N-terminus and central part of α-Syn participate in the helix-rich intermediate formation, while C-terminus remains flexible in this state. The Trp fluorescence and quenching studies reveal that site-specific structural dynamics at the N-terminus, NAC region and C-terminus are involved in the formation of helix-rich state; however most drastic transition at the NAC region mediate the helix maturation into fibrils. It is suggested that N-terminus of α-Syn is amphipathic in nature and has a propensity to assume helical conformation^87^. It has also been reported that α-Syn binds to membranes *in vitro* forming helix-rich structure composed of its first N-terminal 100 amino acid residue^22^. It is possible that during the initial phase of aggregation, the intermolecular interactions between α-Syn N-terminal segments (having helical propensity) may enhance the stability of the helix conformation (local energy minima). This may further increase the local concentration of α-Syn facilitating intermolecular interactions favoring β-sheet rich amyloid fibril formation.

Since our morphological analysis revealed that the helix-rich intermediate is a heterogeneous population of species, it follows that α-Syn in this state possess multiple structural and oligomeric forms. To characterize the individual components of this helix-rich state, centrifugation was performed. It was observed that ~25% of the helix-rich intermediate are pelletable fibrils having β-sheet conformation; whereas another ~25% soluble fraction are α-Syn species having <100 kDa mass and of random coil conformation. Rest ~50% are of helical conformation having >100 kDa mass. The isolated helix-rich state is a homogeneous population of small oligomeric species, which alone can self-assemble into β-sheet rich fibrils instantaneously without any lag phase suggesting that the helix-rich intermediate is the on-pathway intermediate for fibril formation. However, the morphology of the fibril formed from helix-rich state alone showed thin and short fibrils (even after long incubation) in contrast to long fibrillar network that matured from native helix-rich state (mixture). The data suggest that during the elongation phase, different α-Syn species formed in the nucleation phase might interact with each other differently resulting in fibrils having distinct morphologies. To probe this, we incubated isolated helix-rich state with 10% fibrils and the data showed the formation of long fibrils (morphologically similar to the fibrils matured from LMW). Interestingly, our cell toxicity assays indicate that these helix-rich states of α-Syn are more cytotoxic compared to soluble and fibrillar α-Syn. This high cytotoxicity of helix-rich states could be attributed to their higher exposed hydrophobic surfaces as suggested for oligomeric protein/peptides^65^,^66^.

We propose that the N-terminal regions of α-Syn, which has a high propensity to assume helical structure, might mediate the slow formation of helix-rich intermediate through intermolecular interactions. However, this intermediate is formed in a low amount initially and, therefore, remains undetectable in the lag phase. The intermolecular helix-helix interactions further facilitate aggregation and once small amount of fibrils are formed; they may further auto catalyze the α-Syn aggregation into fibrils, which may also involve the helix-rich intermediate formation. Thus large amount of helix-rich state accumulates at the beginning of elongation phase (25%) and eventually most of the protein species get converted to the thermodynamically favorable state of β-sheet-rich amyloid fibrils at mid elongation phase of aggregation. Moreover, during the elongation phase, the helix-rich state may further interact with preformed fibrils for their further conversion into β-sheet-rich fibrils. Overall our data indicate that both nucleation and elongation phases comprise of a complex aggregation reaction involving the interaction of multiple species, which may be the responsible for the formation of morphologically distinct amyloid fibrils. The present study, therefore, provides valuable insights into the complex aggregation mechanism and extensive characterization of the intermediates involved in the amyloid forming pathway of α-Syn. This study would also contribute in providing targets for therapeutics against Parkinson's disease and other neurodegenerative diseases where "α-helix-to-β-sheet" transitions and α-helix-rich intermediates are linked to disease pathogenesis^41^,^42^,^46^.

## Methods

### Chemicals and Reagents

Chemicals were obtained from Sigma Chemical Co. (St. Louis, MO) and were of the highest purity available. Water was double distilled and deionized using a Milli-Q system (Millipore Corp., Bedford, MA). Cell culture stuffs were purchased from Invitrogen (U.S.A.).

### Multiple sequence alignment

For multiple sequence alignment ClustalW (http//ebi.ac.uk/clustalw2) was used for all synucleins. The input sequences were in FASTA format and the sequences were aligned using the default setting parameters.

### Protein expression and purification

Site-specific Trp mutants were created by site-directed mutagenesis as described by Sahay *et al*^88^. All synucleins were expressed in *Escherichia coli* BL21 (DE3) strain according to the protocol described by Volles *et al*^89^ with slight modification^55^,^90^,^91^.

### Isolation of LMW α-Syn

Lyophilized protein was dissolved in 20 mM Gly-NaOH, pH 7.4, 0.01% sodium azide at a concentration 10 mg/ml. Since synucleins are acidic in nature, the pH of the resulting solution was around 6.0 and it was not fully soluble. To solubilize, few μl of 2 mM NaOH was added until the solution became completely clear. The pH was adjusted to 7.4 by adding few μl of 1 M HCl. The pH of the final solution was confirmed by micro pH meter (S20 Seven Easy, Mettler-Toledo, Switzerland). The solution was then dialyzed in a mini dialysis unit of 10 kDa cutoff (Slide-A-Lyzer Mini dialysis devices, Pierce, USA) for overnight against the same buffer. For (30−110) α-Syn, 3.5 kDa mini dialysis units were used. The solutions were then collected, transferred to filters (100,000 molecular weight cut-off (MWCO) Centricon YM-100, Millipore Corp., Bedford, MA). For (30−110) α-Syn, the solution was transferred to YM-50. Then filters were centrifuged for 30 min at 10,000 × g using a benchtop microcentrifuge (Eppendorf Model 5415C, Brinkmann Instruments Inc., Westbury, NY). The resulting solution was clear and free of any larger aggregates. The cutoff filters were washed three times with buffer before use. α-Syn was shown to contain monomeric α-Syn in equilibrium with low-order, unstructured oligomers. The concentrations of synucleins in the filtrates were determined by absorbance at 280 nm, considering the molar absorptivity (ε) is 5960 for α-Syn, its three disease mutants, mouse-Syn, β-Syn and 1490 for [30−110] α-Syn and γ-Syn.

### Amyloid fibril formation

The assembly reaction was initiated with LMW synuclein at a concentration of ~300 μM in 1.5 ml eppendorf tube in 20 mM Gly-NaOH, pH 7.4, with 0.01% sodium azide. For morphological analysis of α-Syn species during aggregation, aggregation reaction of WT LMW α-Syn at 250 μM concentration in 20 mM Gly-NaOH buffer, pH 7.4, 0.01% sodium azide at 37 °C with slight agitation (~50 r.p.m) was initiated. The eppendorf tubes containing protein solutions were placed into an Echo Therm model RT11 rotating mixture (Torrey Pines Scientific, USA) with a speed corresponding to 50 r.p.m. inside a 37°C incubator. The fibril formation was monitored by CD, ThT binding and confirmed by EM at the end of assembly reaction.

### ThT fluorescence assay

1 mM ThT was prepared in Tris-HCl buffer, pH 8.0, 0.01% sodium azide. 2 μl of 1 mM ThT solution was added to the 7.5 μM protein solution in 200 μL Gly-NaOH buffer, pH 7.4, 0.01% sodium azide. Immediately after addition, ThT fluorescence assay was done using Horiba-Jobin Yvon (Fluomax4) with excitation at 450 nm and emission in the range of 465−500 nm. The slit width for both excitation and emission were kept at 5 nm. ThT fluorescence obtained at 480 nm was plotted for all proteins against incubation time and the data were fitted to a sigmoidal curve. The lag time (t_lag_) was calculated according to the published protocol^56^ using equation y = y_0_ + (y_max__−_ y_0_)/(1 + e-^(k(t-t^_1/2_^)^), where y is the ThT fluorescence at a particular time point, y_max_ is the maximum ThT fluorescence and y_0_ is the ThT fluorescence at t_0_ and t_lag_ was defined as t _lag_ = t _½_ − 2/k.

### Circular dichroism spectroscopy (CD)

5 μL of protein solution was diluted to 200 μl in 20 mM Gly-NaOH, pH 7.4 with 0.01% sodium azide. The solution was placed into a 0.1 cm path-length quartz cell (Hellma, Forest Hills, NY). Spectra were acquired using JASCO-810 instrument. All measurements were done at 25°C. Spectra were generally recorded over the wavelength range of 198−260 nm. Each spectrum was scanned thrice (accumulation 3) and the average was taken. Raw data were processed by smoothing and subtraction of buffer spectra, according to the manufacturer's instructions. Three independent experiments were performed with each sample. Spectral deconvolution of CD data was performed using the CDPro software^57^ package that consisted of three programs (viz. SELCON3, CDSSTR, and CONTINLL) to determine relative quantities of random coil, α-helix, β-sheet and β-turn. In the CDPro package, reference sets of proteins from different sources are combined to create a large reference set of CD spectra and depending upon the spectrum wavelength range of the experiment, the reference set (*IBasis*) can be changed. We used either an *IBasis* of 7 (48 proteins reference set) or *IBasis* of 9 (50 protein reference set), depending upon the curve fitting with experimental CD spectra that produced lower RMSD value. Wavelength was taken form (240−198) nm for the deconvolution for all three programs in CDPro. If the data obtained were similar from the three different programs, they were averaged to obtain the percentage of each secondary structure element. In some cases, the results obtained from one program were not consistent with those from the other two. Such cases averaging were done with data set from two programs that produced similar results.

### Atomic force microscopy (AFM)

For atomic force microscopy, protein samples were spotted on a freshly cleaved mica sheet followed by washing with double distilled water. Immediate after addition, mica was dried in vacuum desiccator. AFM imaging was done in tapping mode under a silicon nitride cantilever using Veeco Nanoscope IV Multimode AFM. Minimum five different areas of three independent samples were scanned with a scan rate of 1.5 Hz.

### Electron microscopy (EM)

The aged synuclein solutions in 20 mM Gly-NaOH, pH 7.4, 0.01% sodium azide were diluted in distilled water to a concentration of ~40 μM, spotted on a glow-discharged, carbon-coated Formvar grid (Electron Microscopy Sciences, Fort Washington, PA), incubated for 5 min, washed with distilled water, and then stained with 1% (w/v) aqueous uranylformate solution. Uranylformate solution was prepared freshly and filtered through 0.22 μm sterile syringe filters (Milipore, USA). EM analysis was performed using an electron microscope (FEI Tecnai G2 12) at 120 kV with nominal magnifications in the range of 26,000 to 60,000. Images were recorded digitally by using the SIS Megaview III imaging system. At least two independent experiments were carried out for each sample.

### Tryptophan fluorescence

LMW proteins were prepared for all Trp mutants and WT protein and the concentrations of protein were determined by measuring absorbance at 280 nm considering the molar absorptivity (ε) as 11460 M^−1^cm^−1^ for single tryptophan mutant of WT α-Syn. The LMW of Trp mutants and WT α-Syn at 300 μM concentrations in 20 mM Gly-NaOH buffer, pH 7.4, 0.01% sodium azide were incubated with slight agitation at 37°C. During incubation, small aliquots of the samples were taken at regular intervals and diluted with identical buffer with final protein concentration of 6 μM for Trp fluorescence and 6 μM for CD spectroscopy. Both CD and Trp fluorescence spectroscopy were performed simultaneously. Fluorescence spectra were recorded using Horiba Jobin Yvon (Fluoromax 4) with excitation at 290 nm and emission in the range 300−500 nm. The excitation as well as emission slit widths were set to 5 nm. To monitor the site specific conformational changes during aggregation, the emission maxima (λ_max_) was plotted against the time course of incubation. Further, λ_max_ of Trp emission spectra was determined using gnu plot fitted to a second order polynomial equation. For that the value of the Trp emission spectra from 335 to 375 was taken and fitted using the function f(x) = −a(x − b)^2^ + C; Where ‘b' corresponds to λ_max_ for the corresponding Trp emission spectra.

### Acrylamide quenching

300 μM LMW of Trp mutants of WT α-Syn in 20 mM Gly-NaOH buffer, pH 7.4, 0.01% sodium azide were incubated with slight agitation (~50 r.p.m) at 37 °C. The CD study was performed at regular intervals to monitor the aggregation as described in Trp fluorescence section. Site-specific solvent exposures of Trp of α-Syn Trp mutants in LMW, helical intermediate and β-sheet-rich state were performed using acrylamide quenching. For the quenching experiments, aliquots of the protein solutions were mixed with increasing concentrations of acrylamide (0−0.5 M) where final concentration of protein was 6 μM in each preparation. The solutions were incubated in the dark for five minutes. Fluorescence spectra were recorded with excitation at 290 nm and emission in the range of 300−500 nm. The fluorescence intensity maxima in absence (F_0_) and presence of different acrylamide concentrations (F) were plotted against different acrylamide concentrations and these plots were fitted using the modified Stern-Volmer equation^60^; F_0_/F = (1 + K_sv_[Q])e^v[Q]^; where K_sv_ is the Stern-Volmer constant, [Q] is the acrylamide concentration, V is the static quenching constant. These fitting yield Stern-Volmer constants, which were plotted against different species studied.

### NMR Spectroscopy

All NMR experiments were performed on a Bruker Avance 800 MHz NMR spectrometer equipped with triple resonance gradient probe at 288 K. Data were processed using Topspin 2.1 version and analyzed with Sparky 3.114. Spectra of LMW and helical intermediates of α-Syn were collected at a concentration of ~240 μM in the H_2_O/D_2_O (90:10) in Gly-NaOH buffer, pH 7.4. Backbone resonance assignments of LMW wt α-Syn were obtained using standard set of triple resonance experiments (such as HNN, CBCANH, CBCACONH, HNCO) together with previously published data (BMRB accession number 16543 and 5744). For calculating H^α^ secondary chemical shift, 3D TOCSY-HSQC experiments with MLEV-17 sequence and with a mixing time of 60 ms were recorded. To obtain various through-space connection in helical intermediates, 3D NOESY-HSQC spectra, with mixing time of 200 ms, were recorded. All 2D experiments were acquired with 8−32 scans having (2048 × 256) data matrix. The data were zero-filled to give a (4096 × 1024) matrix and processed prior to Fourier transformation with sine square bell. All proton chemical shifts were referenced with using DSS as an external reference and other nuclei were reference indirectly using BMRB protocol.

### Isolation of helical intermediate

To isolate the helical intermediate from the reaction mixture, 300 μM LMW of α-Syn was prepared as described earlier^54^,^55^ and incubated at 37°C with slight agitation (~50 r.p.m). Its secondary structural changes were monitored by CD spectroscopy. When helical conformation appeared in CD measurement (two negative minima one at ~205−208 nm and another at ~222 nm), the reaction mixture was taken out from the incubator. It was centrifuged at 14000 g for 15 minutes at 4°C. This leads to the separation of pelletable fibrils (P) and supernatant (S). The supernatant was passed through 100 kDa cut off filter. The flow-through (F, MW less than 100 kDa) and the retentate (R, upper portion of cutoff filter (more than 100 kDa)) were collected. From 300 μM reaction mixture, ~60 μM pellet, ~80 μM flow-through and ~160 μM isolated helix were obtained.

### Aggregation of synuclein in presence of isolated helix

300 μM LMW (<100 kDa) solution of α-Syn in 20 mM Gly-NaOH, pH 7.4, 0.01% sodium azide was incubated at 37°C with slight agitation (50 r.p.m) and the aggregation kinetics was monitored by CD. When solution showed helix-rich conformation in CD spectroscopy, the protein solution was taken out from the incubator and helix was isolated using 100 kDa cutoff filter as described previously. Aliquots of this solution were added to freshly prepared LMW α-Syn such that helical seeds become 10% (v/v). Only LMW α-Syn was also used as a control. Similar experiments also were performed with preformed fibrils as seeds instead of helical intermediate for the positive control. For seeding study with helix-rich intermediate and fibrils, the protein concentration, and total volume were kept constant with 300 μM and 400 μl, respectively. All solution mixture was incubated at 37°C with slight agitation and during incubation ThT binding was measured at regular time intervals. ThT was measured using spectrofluorometer Horiba Jobin Yvon (Fluoromax-4) with excitation at 450 nm and emission in the range 460−500 nm. Both excitation and emission slit widths used for the fluorescence measurement was 5 nm.

### FTIR study

FTIR is a powerful technique for the determination individual secondary structural components of protein/peptides. For FTIR analysis, thin translucent pellet of KBr was made by compressing the grounded KBr powder at the pressure of 7 ton by using hydraulic pressure pump. The pellet was then kept under IR lamp and 5 μl of the α-Syn solution was spotted on it and dried immediately. For background spectrum 5 μl of the buffer was spotted on another KBr pellet and dried. The pellet was then kept in a transmission holder and the IR spectra in the range of 1800−1500 cm^−1^ were acquired by using BrukerVertex-80 instrument equipped with DTGS detector. For each spectrum, 32 scans at the resolution of 2 cm^−1^ were recorded. Raw data corresponding to amide-I region (1700−1600 cm^−1^) were deconvoluted by Fourier self-deconvolution (FSD) method. The deconvoluted spectra were then subjected to Lorentzian curve fitting procedure and integrated using opus-65 software according to manufactures instruction.

### ANS binding assay

1-anilinonaphthalene-8-sulphonic acid (ANS) is a powerful dye to measure the exposed hydrophobic surfaces of protein and its aggregates^65^,^66^,^67^. ANS binding study was performed during aggregation of WT α-Syn. To determine the ANS binding of the helix-rich intermediate, 300 μM freshly solubilized WT α-Syn in 20 mM Gly-NaOH, pH 7.4 was incubated at 37°C with slight agitation. When the helical intermediate appeared in CD measurement, it was isolated as described previously. 3 μl of 5 mM ANS was added to 5 μl of different species of α-Syn in 200 μl buffer. It was incubated for 10 min at dark. ANS fluorescence was measured using Horiba Jobin Yvon (Fluoromax 4) spectrofluorometer exciting at 370 nm and emission in the range of 400−600 nm with excitation and emission slit widths were of 5 nm. Buffer subtraction was done and fluorescence intensity at 475 nm was plotted against different synuclein species.

### MTT assay

LMW wt α-Syn at 300 μM concentration was incubated at 37°C with slight agitation. When the helical intermediate was detected in CD, helix was isolated as described previously. Fibrils and freshly prepared LMW were also used as controls. Subsequently, similar toxicity assay were also performed with other mixture assembly containing of helix-rich conformation. To do the toxicity, neuronal cell line of SH-SY5Y were cultured in Dulbecco's Modified Eagle Medium (DMEM) (Himedia, India) supplemented with 10% FBS (Invitrogen, USA), 100 units/mL penicillin and 100 μg/mL streptomycin in a 5% CO_2_ humidified environment at 37°C. Cells were seeded in 96-well plates in 100 μL medium at a cell density of ~10,000 per well. After 24 hr of incubation, the old medium was replaced with fresh media containing 50 μM of each α-Syn species. Cells were further incubated for 24 hr at 37°C. After this, 10 μl of a 5 mg/ml MTT prepared in PBS was added to each well and the incubation was continued for 4 hr. Finally, 100 μl of a solution containing 50% dimethylformamide and 20% SDS (pH 4.7) was added and incubated for overnight. After incubation in a 5% CO_2_ humidified environment at 37°C, absorption values at 560 nm were determined with an automatic micro titer plate reader (Thermo Fisher Scientific, USA). The background absorbance was also recorded at 690 nm and subtracted from the absorbance value of 560 nm.

### Cell viability assay

To check the cell viability in the presence of various α-Syn species (LMW, helical intermediates, and fibrils) calcein and ethidium homodimer 1 staining was used. To do that different species of α-Syn were isolated as described previously. For cell culture, SHSY-5Y cells were cultured on glass cover slips of 12 mm diameter with a density of 4 × 10^4^. The cells were maintained in DMEM (HIMEDIA, India) with 10% heat-inactivated FBS and 100 units/ml penicillin and 100 μg/ml streptomycin in a 37°C incubator with a humidified atmosphere of 5% CO_2_ for 24 hrs. Next day, the old culture media was replaced with fresh media along with the addition of various α-Syn species at a concentration of 10 μM per well and cells were further incubated for 22 hours at 37°C. After this incubation, cells were washed once with PBS and then treated with 2 mM calcein and 4 mM of ethidium homodimer 1 simultaneously for 10 mins at 37°C. After removal of staining solutions, cell were washed once with PBS and fixed in 4% PFA, which then mounted for imaging under fluorescence microscope (Axiocam, Zeiss).

### Cell morphology analysis

To study the effect of various α-Syn species on the cell morphology, neuroblastoma cell lines SH-SY 5Y, were seeded at a density of 4 × 10^4^ on the coverslips and maintained for 24 hrs as previously mentioned. Next day, the old culture media was replaced with fresh media along with the addition of various α-Syn species at a concentration of 10 μM per well and cells were further incubated for 22 hours at 37°C. Further, the cells were fixed in 4% PFA then taken for imaging using phase contrast microscopy (X 51, Olympus).

### Fluorocytometry analysis

For relative quantification of cell death and apoptosis in the presence of helix-rich intermediate, flow cytometry measurement was performed using Annexin V-FITC apoptosis detection kit (Sigma, USA). To do that, undifferentiated cells were grown in T25 cell culture flask (Nunc, USA) until ~70% confluency (~10^6^ cells). The cells were then treated each with 50 μM each of helix-rich intermediate, LMW and fibrils and only buffer for 24 hrs. After incubation, the cells were trypsinized, centrifuged and used for cell death assay using Annexin V-FITC Apoptosis detection kit (APOAF, Sigma, USA). The cell pellet was washed with 1 × PBS and further resuspended in 1 × binding buffer (Sigma, USA). Cells were stained with Annexin V-FITC and propidium iodide (PI) according to manufacturer's instructions. Unstained cells (without Annexin V-FITC and PI) were used as a control and cells stained with either Annexin V-FITC or PI was used as fluorescent compensation controls. AnnexinV-FITC and PI staining were quantified in a flow cytometer (FACSAria, BD Biosciences, San Jose, CA) and analyzed using the BDFACS Diva software. For each sample, 20,000 cells were analyzed. The upper left quadrant (Q1) (in Fig. 6E) represents dead cells population (only PI positive), upper right quadrant (Q2) represents the population of cells in the late stage of apoptosis (Annexin V-FITC positive and PI positive), Lower left quadrant (Q3) represents the population of viable/live cells (Annexin V-FITC negative and PI negative), lower right quadrant (Q4) represents the population of cells in early stages of apoptosis (only Annexin V-FITC positive)^72^,^73^. X-axis represents area of Annexin V-FITC fluorescence and Y-axis represents PI fluorescence intensities.

### Statistical analysis

The statistical significance was determined by one-way ANOVA followed by Newman-Keuls Multiple Comparison post hoc test; *P < 0.05; **P < 0.01; NS P > 0.05.

## Author Contributions

S.K.M., R.R., P.K.S. and D.G. designed research. D.G., P.K.S., S.S., N.N.J., R.S.J. and A.K. performed experiment. S.K.M., R.R., A.K., D.G. and S.Sen analyzed data. D.G. prepared figures. S.K.M. and D.G. wrote the manuscript. All authors gave significant inputs on the manuscript discussion. A.K. thanks Ramalingaswamy fellowship for financial support and TIFR for the NMR facility.

## Supplementary Material

Supplementary Information Structure based aggregation studies reveal the presence of helix-rich intermediate during α-Synuclein aggregation

## References

[b1] Clayton D. F. & George J. M. The synucleins: a family of proteins involved in synaptic function, plasticity, neurodegeneration and disease. Trends Neurosci. 21, 249–254 (1998). 9641537 10.1016/s0166-2236(97)01213-7

[b2] Iwai A. *et al.* The precursor protein of non-Aβ component of Alzheimer's disease amyloid is a presynaptic protein of the central nervous system. Neuron 14, 467–475 (1995). 7857654 10.1016/0896-6273(95)90302-x

[b3] Zhang L. *et al.* Semi-quantitative analysis of α-Syn in subcellular pools of rat brain neurons: an immunogold electron microscopic study using a C-terminal specific monoclonal antibody. Brain Res. 1244, 40–52 (2008). 18817762 10.1016/j.brainres.2008.08.067

[b4] Abeliovich A. *et al.* Mice lacking α-Syn display functional deficits in the nigrostriatal dopamine system. Neuron 25, 239–252 (2000). 10707987 10.1016/s0896-6273(00)80886-7

[b5] Cabin D. E. *et al.* Synaptic vesicle depletion correlates with attenuated synaptic responses to prolonged repetitive stimulation in mice lacking α-Syn. J. Neurosci. 22, 8797–8807 (2002). 12388586 10.1523/JNEUROSCI.22-20-08797.2002 PMC6757677

[b6] Volles M. J. & Lansbury P. T. Jr Zeroing in on the pathogenic form of α-Syn and its mechanism of neurotoxicity in Parkinson's disease. Biochemistry 42, 7871–7878 (2003). 12834338 10.1021/bi030086j

[b7] Spillantini M. G. *et al.* α-Syn in Lewy bodies. Nature 388, 839–840 (1997). 9278044 10.1038/42166

[b8] Polymeropoulos M. H. *et al.* Mutation in the α-Syn gene identified in families with Parkinson's disease. Science 276, 2045–2047 (1997). 9197268 10.1126/science.276.5321.2045

[b9] Kruger R. *et al.* Ala30Pro mutation in the gene encoding α-Syn in Parkinson's disease. Nat. Genet. 18, 106–108 (1998). 9462735 10.1038/ng0298-106

[b10] Zarranz J. J. *et al.* The new mutation, E46K, of α-Syn causes Parkinson and Lewy body dementia. Ann. Neurol. 55, 164–173 (2004). 14755719 10.1002/ana.10795

[b11] Singleton A. B. *et al.* α-Syn locus triplication causes Parkinson's disease. Science 302, 841 (2003). 14593171 10.1126/science.1090278

[b12] Chartier-Harlin M. C. *et al.* α-Syn locus duplication as a cause of familial Parkinson's disease. Lancet 364, 1167–1169 (2004). 15451224 10.1016/S0140-6736(04)17103-1

[b13] Lesage S. *et al.* G51D α-Syn mutation causes a novel parkinsonian-pyramidal syndrome. Ann. Neurol. 73, 459–471 (2013). 23526723 10.1002/ana.23894

[b14] Kiely A. P. *et al.* α-synucleinopathy associated with G51D SNCA mutation: a link between Parkinson's disease and multiple system atrophy? Acta. Neuropathol. 125, 753–769 (2013). 23404372 10.1007/s00401-013-1096-7 PMC3681325

[b15] Proukakis C. *et al.* A novel α-Syn missense mutation in Parkinson disease. Neurology 80, 1062–1064 (2013). 23427326 10.1212/WNL.0b013e31828727ba PMC3653201

[b16] Appel-Cresswell S. *et al.* α-Syn p.H50Q, a novel pathogenic mutation for Parkinson's disease. Mov. Disord. 28, 811–813 (2013). 23457019 10.1002/mds.25421

[b17] Feany M. B. & Bender W. W. A Drosophila model of Parkinson's disease. Nature 404, 394–398 (2000). 10746727 10.1038/35006074

[b18] Hashimoto M., Rockenstein E. & Masliah E. Transgenic models of α-Syn pathology: past, present, and future. Ann. N. Y. Acad. Sci. 991, 171–188 (2003). 12846986

[b19] Uversky V. N. *et al.* Biophysical properties of the synucleins and their propensities to fibrillate: inhibition of α-Syn assembly by β- and γ-Syn. J. Biol. Chem. 277, 11970–11978 (2002). 11812782 10.1074/jbc.M109541200

[b20] Giasson B. I., Murray I. V., Trojanowski J. Q. & Lee V. M. A hydrophobic stretch of 12 amino acid residues in the middle of α-Syn is essential for filament assembly. J. Biol. Chem. 276, 2380–2386 (2001). 11060312 10.1074/jbc.M008919200

[b21] Recchia A. *et al.* α-Syn and Parkinson's disease. FASEB J 18, 617–626 (2004). 15054084 10.1096/fj.03-0338rev

[b22] Eliezer D., Kutluay E., Bussell R. Jr & Browne G. Conformational properties of α-Syn in its free and lipid-associated states. J. Mol. Bio. 307, 1061–1073 (2001). 11286556 10.1006/jmbi.2001.4538

[b23] Lee S. J., Jeon H. & Kandror K. V. α-Syn is localized in a subpopulation of rat brain synaptic vesicles. Acta. Neurobiol. Exp. (Wars) 68, 509–515 (2008). 19112474 10.55782/ane-2008-1717

[b24] Iyer A., Petersen N. O., Claessens M. M. & Subramaniam V. Amyloids of α-Syn affect the structure and dynamics of supported lipid bilayers. Biophys. J. 106, 2585–2594 (2014). 24940776 10.1016/j.bpj.2014.05.001 PMC4070068

[b25] Sciacca M. F. *et al.* Two-step mechanism of membrane disruption by Aβ through membrane fragmentation and pore formation. Biophys. J. 103, 702–710 (2012). 22947931 10.1016/j.bpj.2012.06.045 PMC3443794

[b26] Sciacca M. F. *et al.* Cations as switches of amyloid-mediated membrane disruption mechanisms: calcium and IAPP. Biophys. J. 104, 173–184 (2013). 23332070 10.1016/j.bpj.2012.11.3811 PMC3540246

[b27] Brender J. R., Salamekh S. & Ramamoorthy A. Membrane disruption and early events in the aggregation of the diabetes related peptide IAPP from a molecular perspective. Acc. Chem. Res. 45, 454–462 (2012). 21942864 10.1021/ar200189b PMC3272313

[b28] Nanga R. P., Brender J. R., Xu J., Veglia G. & Ramamoorthy A. Structures of rat and human islet amyloid polypeptide IAPP(1–19) in micelles by NMR spectroscopy. Biochemistry 47, 12689–12697 (2008). 18989932 10.1021/bi8014357 PMC2953382

[b29] Lorenzen N., Lemminger L., Pedersen J. N., Nielsen S. B. & Otzen D. E. The N-terminus of α-Syn is essential for both monomeric and oligomeric interactions with membranes. FEBS. Lett. 588, 497–502 (2014). 24374342 10.1016/j.febslet.2013.12.015

[b30] Anderson V. L., Ramlall T. F., Rospigliosi C. C., Webb W. W. & Eliezer D. Identification of a helical intermediate in trifluoroethanol-induced α-Syn aggregation. Proc. Natl. Acad. Sci. U. S. A. 107, 18850–18855 (2010). 20947801 10.1073/pnas.1012336107 PMC2973859

[b31] Ahmad M. F., Ramakrishna T., Raman B. & Rao Ch M. Fibrillogenic and non-fibrillogenic ensembles of SDS-bound human α-Syn. J. Mol. Biol. 364, 1061–1072 (2006). 17054982 10.1016/j.jmb.2006.09.085

[b32] Nanga R. P. *et al.* Three-dimensional structure and orientation of rat islet amyloid polypeptide protein in a membrane environment by solution NMR spectroscopy. J. Am. Chem. Soc. 131, 8252–8261 (2009). 19456151 10.1021/ja9010095 PMC4163022

[b33] Ferreon A. C. & Deniz A. A. α-Syn multistate folding thermodynamics: implications for protein misfolding and aggregation. Biochemistry 46, 4499–4509 (2007). 17378587 10.1021/bi602461y

[b34] Kotler S. A., Walsh P., Brender J. R. & Ramamoorthy A. Differences between amyloid β aggregation in solution and on the membrane: insights into elucidation of the mechanistic details of Alzheimer's disease. Chem. Soc. Rev. 43, 6692–6700 (2014). 24464312 10.1039/c3cs60431d PMC4110197

[b35] Vivekanandan S., Brender J. R., Lee S. Y. & Ramamoorthy A. A partially folded structure of amyloid-β_1−40_ in an aqueous environment. Biochem. Biophys. Res. Commun. 411, 312–316 (2011). 21726530 10.1016/j.bbrc.2011.06.133 PMC3148408

[b36] Esposito V., Das R. & Melacini G. Mapping polypeptide self-recognition through ^1^H off-resonance relaxation. J. Am. Chem. Soc. 127, 9358–9359 (2005). 15984849 10.1021/ja051714i

[b37] Milojevic J., Raditsis A. & Melacini G. Human serum albumin inhibits Aβ fibrillization through a “monomer-competitor” mechanism. Biophys. J. 97, 2585–2594 (2009). 19883602 10.1016/j.bpj.2009.08.028 PMC2770600

[b38] Ramamoorthy A. & Lim M. H. Structural characterization and inhibition of toxic amyloid β oligomeric intermediates. Biophys. J. 105, 287–288 (2013). 23870249 10.1016/j.bpj.2013.05.004 PMC3714932

[b39] Milojevic J. & Melacini G. Stoichiometry and affinity of the human serum albumin-Alzheimer's Aβ peptide interactions. Biophys. J. 100, 183–192 (2011). 21190670 10.1016/j.bpj.2010.11.037 PMC3010178

[b40] Apetri M. M., Maiti N. C., Zagorski M. G., Carey P. R. & Anderson V. E. Secondary structure of α-Syn oligomers: characterization by raman and atomic force microscopy. J. Mol. Biol. 355, 63–71 (2006). 16303137 10.1016/j.jmb.2005.10.071

[b41] Kirkitadze M. D., Condron M. M. & Teplow D. B. Identification and characterization of key kinetic intermediates in amyloid β protein fibrillogenesis. J. Mol. Biol. 312, 1103–1119 (2001). 11580253 10.1006/jmbi.2001.4970

[b42] Williamson J. A. & Miranker A. D. Direct detection of transient α-helical states in islet amyloid polypeptide. Protein. Sci. 16, 110–117 (2007). 17123962 10.1110/ps.062486907 PMC2222845

[b43] Yonemoto I. T., Kroon G. J., Dyson H. J., Balch W. E. & Kelly J. W. Amylin proprotein processing generates progressively more amyloidogenic peptides that initially sample the helical state. Biochemistry. 47, 9900–9910 (2008). 18710262 10.1021/bi800828u PMC2662778

[b44] Liu G. *et al.* Mechanistic studies of peptide self-assembly: transient α-helices to stable β-sheets. J. Am. Chem. Soc. 132, 18223–18232 (2010). 21138275 10.1021/ja1069882

[b45] Meng F., Abedini A., Song B. & Raleigh D. P. Amyloid formation by pro-islet amyloid polypeptide processing intermediates: examination of the role of protein heparan sulfate interactions and implications for islet amyloid formation in type 2 diabetes. Biochemistry 46, 12091–12099 (2007). 17924651 10.1021/bi7004834

[b46] Abedini A. & Raleigh D. P. A role for helical intermediates in amyloid formation by natively unfolded polypeptides? Phys. Biol. 6, 015005 (2009). 19208933 10.1088/1478-3975/6/1/015005 PMC3215505

[b47] Williamson J. A., Loria J. P. & Miranker A. D. Helix stabilization precedes aqueous and bilayer-catalyzed fiber formation in islet amyloid polypeptide. J. Mol. Biol. 393, 383–396 (2009). 19647750 10.1016/j.jmb.2009.07.077 PMC3343364

[b48] Hauser C. A. *et al.* Natural tri- to hexapeptides self-assemble in water to amyloid β type fiber aggregates by unexpected α-helical intermediate structures. Proc. Natl. Acad. Sci. U. S. A. 108, 1361–1366 (2011). 21205900 10.1073/pnas.1014796108 PMC3029732

[b49] Andreola A. *et al.* Conformational switching and fibrillogenesis in the amyloidogenic fragment of apolipoprotein a-I. J. Biol. Chem. 278, 2444–2451 (2003). 12421824 10.1074/jbc.M204801200

[b50] Klimov D. K. & Thirumalai D. Dissecting the assembly of Aβ_16-22_ amyloid peptides into antiparallel β sheets. Structure. 11, 295–307 (2003). 12623017 10.1016/s0969-2126(03)00031-5

[b51] Nerelius C. *et al.* α-helix targeting reduces amyloid β peptide toxicity. Proc. Natl. Acad. Sci. U. S. A. 106, 9191–9196 (2009). 19458258 10.1073/pnas.0810364106 PMC2695042

[b52] Wood S. J. *et al.* α-synuclein fibrillogenesis is nucleation-dependent. Implications for the pathogenesis of Parkinson's disease. J. Biol. Chem. 274, 19509–19512 (1999). 10391881 10.1074/jbc.274.28.19509

[b53] LeVine H. 3rd Quantification of β-sheet amyloid fibril structures with thioflavin T. Methods. Enzymol. 309, 274–284 (1999). 10507030 10.1016/s0076-6879(99)09020-5

[b54] Winner B. *et al.* In vivo demonstration that α-Syn oligomers are toxic. Proc. Natl. Acad. Sci. U. S. A. 108, 4194–4199 (2011). 21325059 10.1073/pnas.1100976108 PMC3053976

[b55] Singh P. K. *et al.* Curcumin modulates α-Synuclein aggregation and toxicity. ACS. Chem. Neurosci. 4, 393–407 (2013). 23509976 10.1021/cn3001203 PMC3605819

[b56] Willander H. *et al.* BRICHOS domains efficiently delay fibrillation of amyloid β peptide. J. Biol. Chem. 287, 31608–31617 (2012). 22801430 10.1074/jbc.M112.393157 PMC3438992

[b57] Sreerama N. & Woody R. W. Estimation of protein secondary structure from circular dichroism spectra: comparison of CONTIN, SELCON, and CDSSTR methods with an expanded reference set. Anal. Biochem. 287, 252–260 (2000). 11112271 10.1006/abio.2000.4880

[b58] Sreerama N. & Woody R. W. On the analysis of membrane protein circular dichroism spectra. Protein. Sci. 13, 100–112 (2004). 14691226 10.1110/ps.03258404 PMC2286510

[b59] Barth A. Infrared spectroscopy of proteins. Biochim. Biophys. Acta. 1767, 1073–1101 (2007). 17692815 10.1016/j.bbabio.2007.06.004

[b60] Lakowicz J. R. Principles of fluorescence spectroscopy 2nd edn (Kluwer Academic, 1999).

[b61] Schwarzinger S. *et al.* Sequence-dependent correction of random coil NMR chemical shifts. J. Am. Chem. Soc. 123, 2970–2978 (2001). 11457007 10.1021/ja003760i

[b62] Lorenzen N. *et al.* How epigallocatechin gallate can inhibit α-synuclein oligomer toxicity in vitro. J. Biol. Chem. 289, 21299–21310 (2014). 24907278 10.1074/jbc.M114.554667 PMC4118093

[b63] Abedini A. & Raleigh D. P. A critical assessment of the role of helical intermediates in amyloid formation by natively unfolded proteins and polypeptides. Protein. Eng. Des. Sel. 22, 453–459 (2009). 19596696 10.1093/protein/gzp036 PMC2719502

[b64] Campioni S. *et al.* The presence of an air-water interface affects formation and elongation of α-Syn fibrils. J. Am. Chem. Soc. 136, 2866–2875 (2014). 24460028 10.1021/ja412105t

[b65] Bolognesi B. *et al.* ANS binding reveals common features of cytotoxic amyloid species. ACS. Chem. Biol. 5, 735–740 (2010). 20550130 10.1021/cb1001203

[b66] Campioni S. *et al.* A causative link between the structure of aberrant protein oligomers and their toxicity. Nat. Chem. Biol. 6, 140–147 (2010). 20081829 10.1038/nchembio.283

[b67] Vamvaca K., Vogeli B., Kast P., Pervushin K. & Hilvert D. An enzymatic molten globule: efficient coupling of folding and catalysis. Proc. Natl. Acad. Sci. U. S. A. 101, 12860–12864 (2004). 15322276 10.1073/pnas.0404109101 PMC516485

[b68] Behl C., Davis J. B., Lesley R. & Schubert D. Hydrogen peroxide mediates amyloid β protein toxicity. Cell 77, 817–827 (1994). 8004671 10.1016/0092-8674(94)90131-7

[b69] Bharti A. C., Takada Y., Shishodia S. & Aggarwal B. B. Evidence that receptor activator of nuclear factor (NF)-kappa B ligand can suppress cell proliferation and induce apoptosis through activation of a NF-kappa B-independent and TRAF6-dependent mechanism. J. Biol. Chem. 279, 6065–6076 (2004). 14645259 10.1074/jbc.M308062200

[b70] Wang X. M. *et al.* A new microcellular cytotoxicity test based on calcein AM release. Hum. Immunol. 37, 264–270 (1993). 8300411 10.1016/0198-8859(93)90510-8

[b71] Zhao H. *et al.* Detection and characterization of the product of hydroethidine and intracellular superoxide by HPLC and limitations of fluorescence. Proc. Natl. Acad. Sci. U. S. A. 102, 5727–5732 (2005). 15824309 10.1073/pnas.0501719102 PMC556312

[b72] Marimpietri D. *et al.* Synergistic inhibition of human neuroblastoma-related angiogenesis by vinblastine and rapamycin. Oncogene. 24, 6785–6795 (2005). 16007159 10.1038/sj.onc.1208829

[b73] Vermes I., Haanen C., Steffens-Nakken H. & Reutelingsperger C. A novel assay for apoptosis. Flow cytometric detection of phosphatidylserine expression on early apoptotic cells using fluorescein labelled Annexin V. J. Immunol. Methods. 184, 39–51 (1995). 7622868 10.1016/0022-1759(95)00072-i

[b74] Uversky V. N., Li J. & Fink A. L. Evidence for a partially folded intermediate in α-syn fibril formation. J. Biol. Chem. 276, 10737–10744 (2001). 11152691 10.1074/jbc.M010907200

[b75] Lashuel H. A., Overk C. R., Oueslati A. & Masliah E. The many faces of α-Syn: from structure and toxicity to therapeutic target. Nat. Rev. Neurosci. 14, 38–48 (2013). 23254192 10.1038/nrn3406 PMC4295774

[b76] Konno T. Multistep nucleus formation and a separate subunit contribution of the amyloidgenesis of heat-denatured monellin. Protein. Sci. 10, 2093–2101 (2001). 11567100 10.1110/ps.20201 PMC2374219

[b77] Borbat P., Ramlall T. F., Freed J. H. & Eliezer D. Inter-helix distances in lysophospholipid micelle-bound α-Syn from pulsed ESR measurements. J. Am. Chem. Soc. 128, 10004–10005 (2006). 16881616 10.1021/ja063122l

[b78] Kelly J. W. Amyloid fibril formation and protein misassembly: a structural quest for insights into amyloid and prion diseases. Structure. 5, 595–600 (1997). 9195890 10.1016/s0969-2126(97)00215-3

[b79] Kelly J. W. The alternative conformations of amyloidogenic proteins and their multi-step assembly pathways. Curr. Opin. Struct. Biol. 8, 101–106 (1998). 9519302 10.1016/s0959-440x(98)80016-x

[b80] Teplow D. B. Structural and kinetic features of amyloid β protein fibrillogenesis. Amyloid. 5, 121–142 (1998). 9686307 10.3109/13506129808995290

[b81] Pan K. M. *et al.* Conversion of α-helices into β-sheets features in the formation of the scrapie prion proteins. Proc. Natl. Acad. Sci. U. S. A. 90, 10962–10966 (1993). 7902575 10.1073/pnas.90.23.10962 PMC47901

[b82] Rochet J. C. & Lansbury P. T. Jr Amyloid fibrillogenesis: themes and variations. Curr. Opin. Struct. Biol. 10, 60–68 (2000). 10679462 10.1016/s0959-440x(99)00049-4

[b83] Anoop A. *et al.* Elucidating the role of disulfide bond on amyloid formation and fibril reversibility of somatostatin-14: relevance to its storage and secretion. J. Biol. Chem. 289, 16884–16903 (2014). 24782311 10.1074/jbc.M114.548354 PMC4059132

[b84] Maji S. K. *et al.* Amino acid position-specific contributions to amyloid β protein oligomerization. J. Biol. Chem. 284, 23580–23591 (2009). 19567875 10.1074/jbc.M109.038133 PMC2749133

[b85] Munishkina L. A., Phelan C., Uversky V. N. & Fink A. L. Conformational behavior and aggregation of α-Syn in organic solvents: modeling the effects of membranes. Biochemistry. 42, 2720–2730 (2003). 12614167 10.1021/bi027166s

[b86] Fezoui Y. & Teplow D. B. Kinetic studies of amyloid β protein fibril assembly. Differential effects of α-helix stabilization. J. Biol. Chem. 277, 36948–36954 (2002). 12149256 10.1074/jbc.M204168200

[b87] Vamvaca K., Volles M. J. & Lansbury P. T. Jr The first N-terminal amino acids of α-Syn are essential for α-helical structure formation in vitro and membrane binding in yeast. J. Mol. Biol. 389, 413–424 (2009). 19285989 10.1016/j.jmb.2009.03.021 PMC2801807

[b88] Sahay S., Anoop A., Krishnamoorthy G. & Maji S. K. Site-specific fluorescence dynamics of α-Syn fibrils using time-resolved fluorescence studies: effect of familial Parkinson's disease-associated mutations. Biochemistry. 53, 807–809 (2014). 24450731 10.1021/bi401543z

[b89] Volles M. J. & Lansbury P. T. Jr Relationships between the sequence of α-Syn and its membrane affinity, fibrillization propensity, and yeast toxicity. J. Mol. Biol. 366, 1510–1522 (2007). 17222866 10.1016/j.jmb.2006.12.044 PMC1868670

[b90] Ghosh D. *et al.* The Parkinson's disease-associated H50Q mutation accelerates α-Syn aggregation in vitro. Biochemistry 52, 6925–6927 (2013). 24047453 10.1021/bi400999d

[b91] Ghosh D. *et al.* The Newly Discovered Parkinson's Disease Associated Finnish Mutation (A53E) Attenuates α-Syn Aggregation and Membrane Binding. Biochemistry. 53, 1619–1621 (2014). 10.1021/bi5010365 25268550

