# Supplementary material for: Structure based aggregation studies reveal the presence of helix-rich intermediate during α-Synuclein aggregation
Source: Sci Rep. 2015 Mar 18;5:9228. doi: 10.1038/srep09228 (PMC4363886; doi:10.1038/srep09228)

Supplementary Information

Structure based aggregation studies reveal the presence of helix-rich intermediate during α-Synuclein aggregation.

Dhiman Ghosh[1], Pradeep K. Singh[1], Shruti Sahay[1], Narendra Nath Jha[1], Reeba S. Jacob[1], Shamik Sen[1], Ashutosh Kumar, Roland Riek[2] and Samir K. Maji[1*]

[1]Department of Biosciences and Bioengineering, IIT Bombay, Powai, Mumbai, India 400076

[2] Laboratory for Physical Chemistry, Wolfgang-Pauli-Str. 10, ETH Zurich, CH-8093 Zurich, Switzerland

[*]To whom correspondence should be addressed. Email: samirmaji@iitb.ac.in

Supplementary figure captions

Supplementary Figure 1. List of synucleins studied and Sequence. (A) List of synucleins studied for the aggregation. (B) α-Syn, mouse-Syn, γ-Syn and β-Syn were aligned using ClustalW2. Red color signifies hydrophobic amino acid residues. Blue and magenta stand for acidic and basic amino acids, respectively. Green color codes for hydroxyl and amine groups. "*" represent identical amino acids; ":" and "." represent conserved and semi conserved substitution, respectively.

Supplementary Figure 2. Helix-rich intermediate evident by CD spectroscopy. (A) CD spectra of all synucleins showing structural conversion during aggregation and amyloid formation. Distinct helix-rich intermediates were seen for all synucleins.

Supplementary Figure 3. Appearance of helix rich intermediate during the fibrils growth. Aggregation kinetics measured by ThT binding along with CD studies showing appearance of helix rich intermediate at the onset of elongation phase, which remained till mid-elongation phase.

Supplementary Figure 4. Aggregation kinetics of WT α-Syn in PBS monitored by CD spectroscopy. CD spectra showing structural conversion from RC→β-sheet via α-helical intermediate during aggregation and amyloid formation of WT α-Syn in PBS. Four independent sets were shown. However, set to set variation of aggregation kinetics occurred in PBS.

Supplementary Figure 5. Bar diagram representation for the deconvolution of CD spectra at different conformation. Deconvolution of the CD spectra showing percentage of different secondary structure in freshly prepared LMW state, at maximum helix-rich conformation and after immediate conformation transition to β-sheet.

Supplementary Figure 6. Deconvolution of CD spectra. Deconvolution of CD spectra showing gradual increase in helicity with progression of time. After formation of β-sheet structure helicity decreased drastically.

Supplementary Figure 7. Correlation plot of lag time vs appearance of helix and time of saturation for the different mutants of synucleins. (A) Correlation plot of lag time vs appearance of helix and (B) time of saturation/50% saturation for different mutants of synucleins showing a linear relationship i.e., mutants with a longer lag time showed late appearance of helix and more time for saturation.

Supplementary Figure 8. Concentration dependent secondary structural changes of WT α-Syn. (A) CD spectra of WT α-Syn at three different concentration (600 μM and 1 μM) showing conversion of secondary structure from random coil to β-sheet via α-helical intermediate in a concentration dependent manner. 1 mM α-Syn showed faster conversion followed by 600 μM.

Supplementary Figure 9. Conformational transition and amyloid formation by α-Syn Trp mutants (A) CD spectra showing the secondary structural transformation of RC→Helix→β-sheet by [V^3W] α-Syn, [$V^{71}W$] α-Syn and [$A^{140}W$] α-Syn during fibril formation. (B) EM micrographs of Trp mutants showing amyloid fibril morphology.

Supplementary Figure 10. Changes of Trp fluorescence intensity maxima (λ_{max}) during aggregation by Trp-substituted WT α-Syn. λ_{max} for Trp fluorescence emission spectra was determined by curve fitting to a second order polynomial equation. λ_{max} was plotted against different time of incubation for these three Trp substituted mutants.

Supplementary Figure 11. Fibrils formed from isolated helix and mixture assembly showed similar toxicity. (A) ANS binding spectra of fibrils formed from isolated helix and mixture assembly. (B) MTT and (C) phase contrast images showed similar toxicity of these two different fibrils.

Supplementary Figure 12. Emission spectra of ANS. Emission ANS spectra for different synuclein species. LMW showed least ANS binding with λ_{max} 515 nm. However, ANS binding gradually blue shifted upon binding to ordered structure. Fibrils showed intermediate ANS binding with a λ_{max} 475 nm**.**

Supplementary Figure 13. Schematic representation showing possible mechanism of random coils → α-helix-rich intermediate → β-sheet transition in α-Syn aggregation. Initially, all α-Syn LMW are mostly in an unstructured conformation. During aggregation, amphipathic helix prone region of α-Syn may self-associate to form oligomeric helical intermediate, which represent the local energy minima of aggregation pathway. This helix-helix association may facilitate the β-sheet fibril formation of α-Syn.

Table 1. Deconvolution of CD spectra for different synuclein species with time of incubation.

α-Syn

Time (h)	Helix (%)	β-sheet (%)	Turn (%)	RC (%)
0	1.93	7.5	3.8	86.3
10	9.23	18	11.3	60.1
25	5	11.4	8	75.1
42	6.2	9.05	6.9	77
54	16	14.7	12.7	57
60	27	18.7	18.1	36
65	35	16.6	17	31
70	26	18.5	21	33.9
80	17.6	21	20.2	34
85	7	36.5	23.75	32.5

E46K

Time (h)	Helix (%)	β-sheet (%)	Turn (%)	RC (%)
0	2.3	7	4.6	85.3
15	17.5	25.75	11.35	43
25	18.5	24	14.75	39.6
30	38.4	14.8	13.3	35.3
50	42.3	12.2	16.3	29
55	43	14	15	28.6
60	32.5	23.7	18.5	25
65	14	32.8	24.8	29.1
70	18	30.5	20.5	30.1

A53T

Time (h)	Helix (%)	β-sheet (%)	Turn (%)	RC (%)
0	5.6	11.05	5.15	77.7
15	9.5			
30	17.4	22.4	17.6	42.5
35	32.4	15	14.5	38.6
45	34.1	16	19.7	28.5
60	33	11.9	14.8	41
70	36.7	18.6	18.2	26.9
75	21.3	33.7	21.9	23.1
80	12.2	36.5	24.6	27.2

A30P

Time (h)	Helix (%)	β-sheet (%)	Turn (%)	RC (%)
0	7.3	11.6	10.7	74
50	3.1	9.6	9.7	78.3
160	18.7	19.6	17.4	44.9
180	16.4	24.2	18.9	40.4
210	11	20.8	16.5	54
230	32.23	21.3	17.5	30.5
260	42.4	27	7.4	22.3
285	24	27.8	20.53	27.8
310	32	17.23	18.36	25.8
330	24.3	27	22.1	27.7
350	19.5	32	23.67	26

E57K

Time (h)	Helix (%)	β-sheet (%)	Turn (%)	RC (%)
0	8.75	12.85	7.65	70.65
15	11.45	29.6	18.15	33.4
30	17.8	21.8	15.3	44.3
35	30	13.1	15	40.7
50	42.16	12	16.7	28.9
60	51	15	11	22.56
65	41.5	17.4	16.17	25.1
70	27.56	26.5	21.8	23.9
75	12.2	36.6	24.3	26.1

[30-110] α-Syn

Time (h)	Helix (%)	β-sheet (%)	Turn (%)	RC (%)
0	3.6	9.15	5.6	80.8
4	7.15	21.55	10.65	60.15
7	14.9	23.5	17.5	39.1
12	16	24.5	15.7	43.2
15	20.5	24.03	16.06	38.06
16	18.9	22.3	15.8	41
17	4.9	47.8	19.35	28.1
20	7	43.5	20.25	28.85

Mouse-Syn

Time (h)	Helix (%)	β-sheet (%)	Turn (%)	α-helix (%)
0	5.7	15.4	10.1	68
7	11.7	15.36	11.26	61.63
15	8.7	19.6	13.5	58
20	21.8	14.5	14.7	49.5
22	28.5	14.6	16	41
25	21	17.7	17	45.5
30	16.5	23	21	39
35	2.4	39.5	24.7	33

γ-Syn

Time (h)	Helix (%)	β-sheet (%)	Turn (%)	RC (%)
0	3.5	6.03	5.6	80.2
90	6.5	12.7	8.5	72.4
150	10	14	8.8	69.9
190	5.6	15.3	10.6	67
230	12.8	23	15.43	47.3
270	27.8	19.5	16.7	35
310	30.5	23.5	19	27.5
350	20.8	26.6	20.7	31.5
410	14.8	24.7	22.45	39
430	3	42	25	29

Supplementary Figure 1.

A

**WT α-Synuclein**	**[E46K] α-Synuclein**	**ϒ-Synuclein**
**[A30P] α-Synuclein**	**[E57K] α-Synuclein**	**[30-110] α-Synuclein: amyloid Core forming Sequence**
**[A53T] α-Synuclein**	**β-Synuclein**	**Mouse-Synuclein**

B

```
α-SYN       MDVFMKGLSKAKEGVVAAAEKTKQGVAEAAGKTKEGVLYVGSKTKEGVVHGVATVAEKTK 60
mouse-SYN   MDVFMKGLSKAKEGVVAAAEKTKQGVAEAAGKTKEGVLYVGSKTKEGVVHGVTTVAEKTK 60
β-SYN       MDVFMKGLSMAKEGVVAAAEKTKQGVTEAAEKTKEGVLYVGSKTREGVVQGVASVAEKTK 60
γ-SYN       MDVFKKGFSIAKEGVVGAVEKTKQGVTEAAEKTKEGVMYVGAKTKENVVQSVTSVAEKTK 60
            **** **:* ******.*.*******:*** ******:***:**:*.**:.*::******

α-SYN       EQVTNVGGAVVTGVTAVAQKTVEGAGSIAAATGFVKKDQLG---KNEEGAPQ--EGILED 115
mouse-SYN   EQVTNVGGAVVTGVTAVAQKTVEGAGNIAAATGFVKKDQMG---KGEEGYPQ--EGILED 115
β-SYN       EQASHLGGAVFS-----------GAGNIAAATGLVKREEFPTDLKPEEVAQEAAEEPLIE 109
γ-SYN       EQANAVSEAVVSSVNTVATKTVEEAENIAVTSGVVRKEDLR------PSAPQ------QE 108
            **.. :. **.:            * .**.::*.*:::::           :       :

α-SYN       MPVDPDNEAYEMPSEEGYQDYEPEA 140
mouse-SYN   MPVDPGSEAYEMPSEEGYQDYEPEA 140
β-SYN       PLMEPEGESYEDPPQEEYQEYEPEA 134
γ-SYN       GVASKEKEEVAEEAQSGGD------ 127
                .   *      .:.  :
```

Supplementary Figure 2.

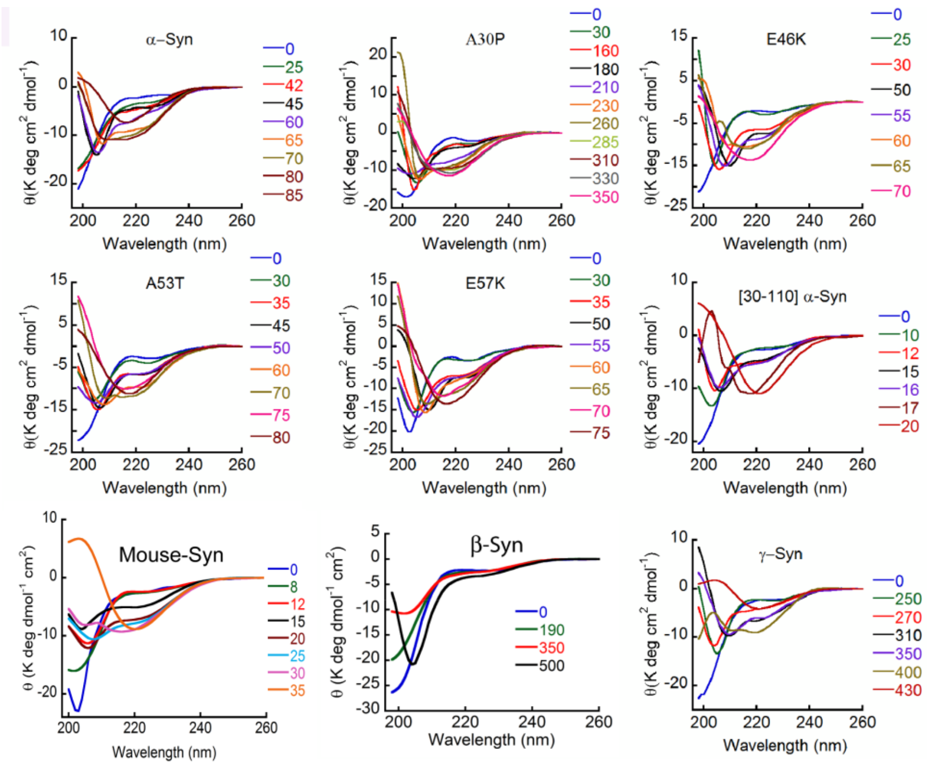

Supplementary Figure 3.

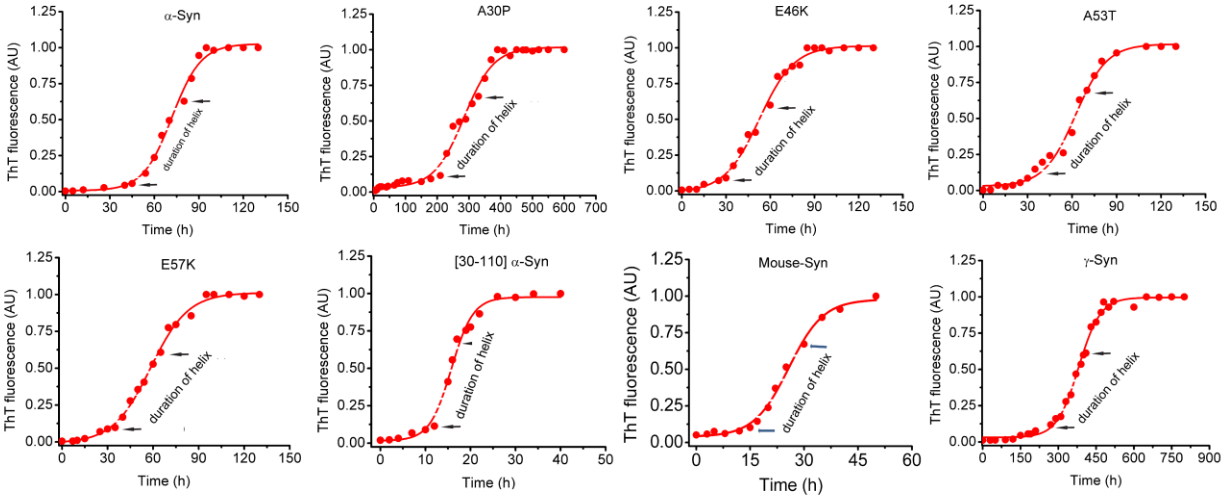

Supplementary Figure 4.

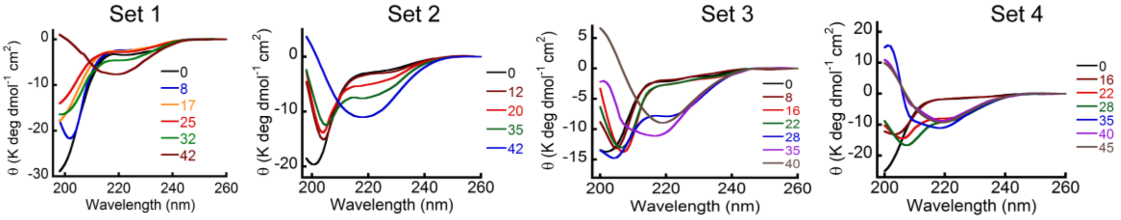

Supplementary Figure 5.

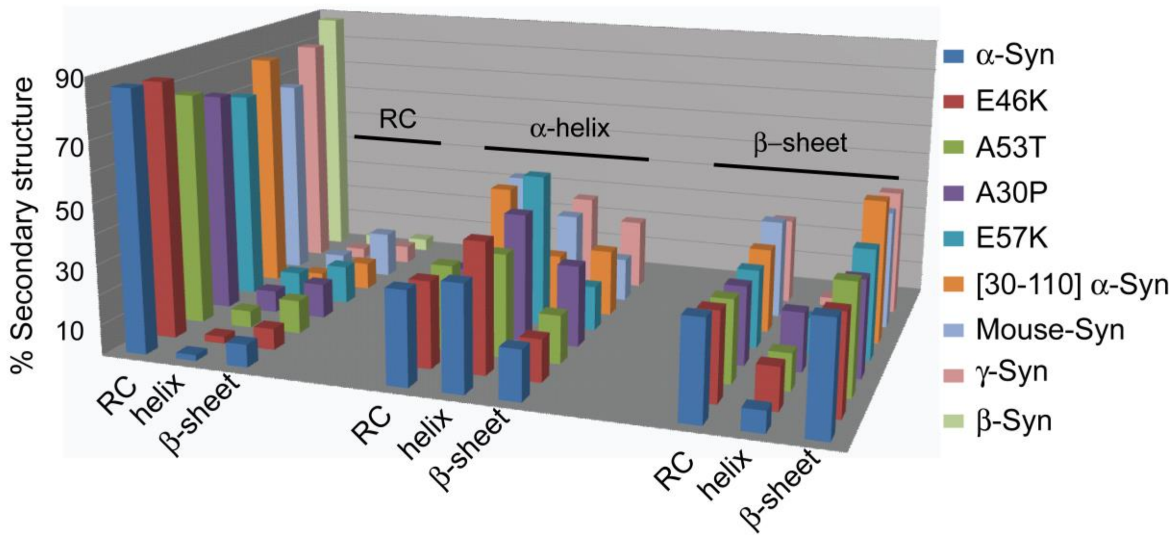

Supplementary Figure 6.

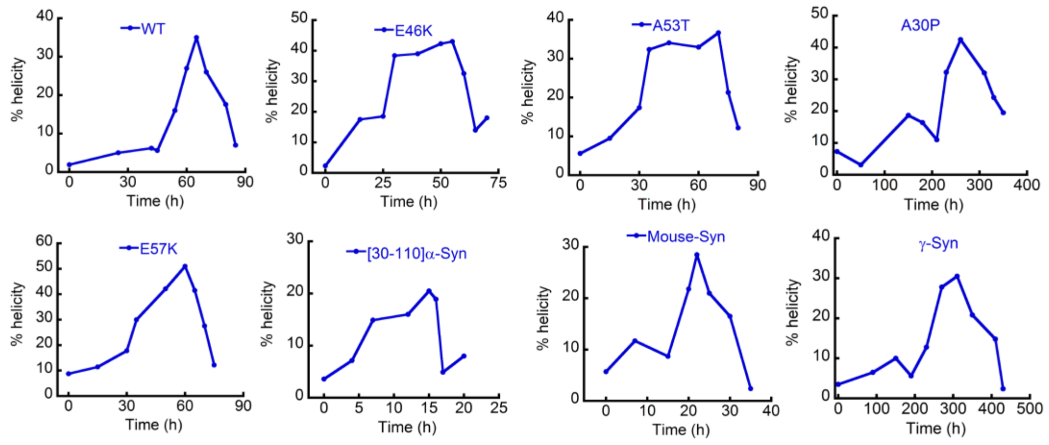

Supplementary Figure 7.

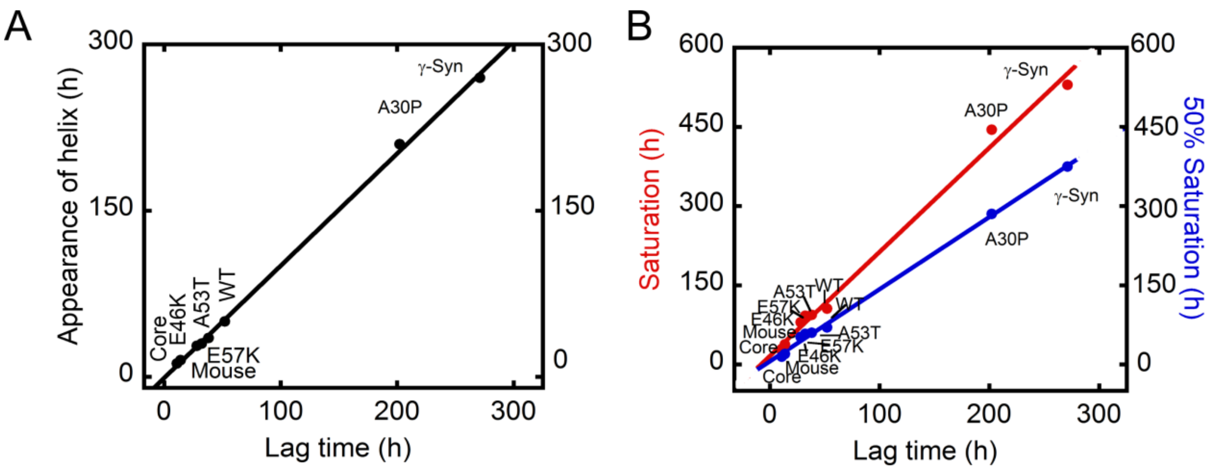

Supplementary Figure 8.

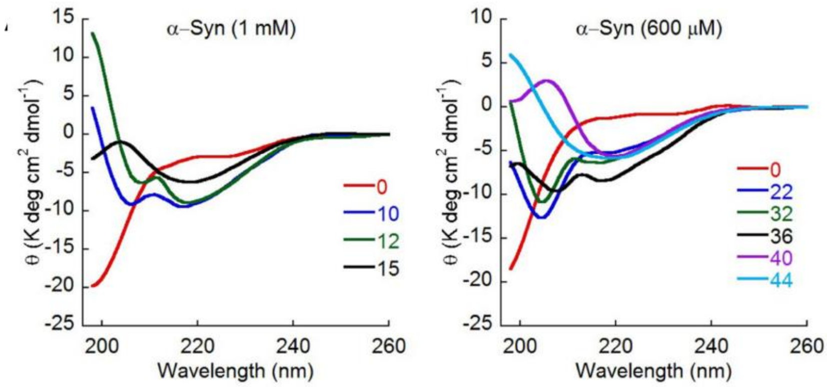

Supplementary Figure 9.

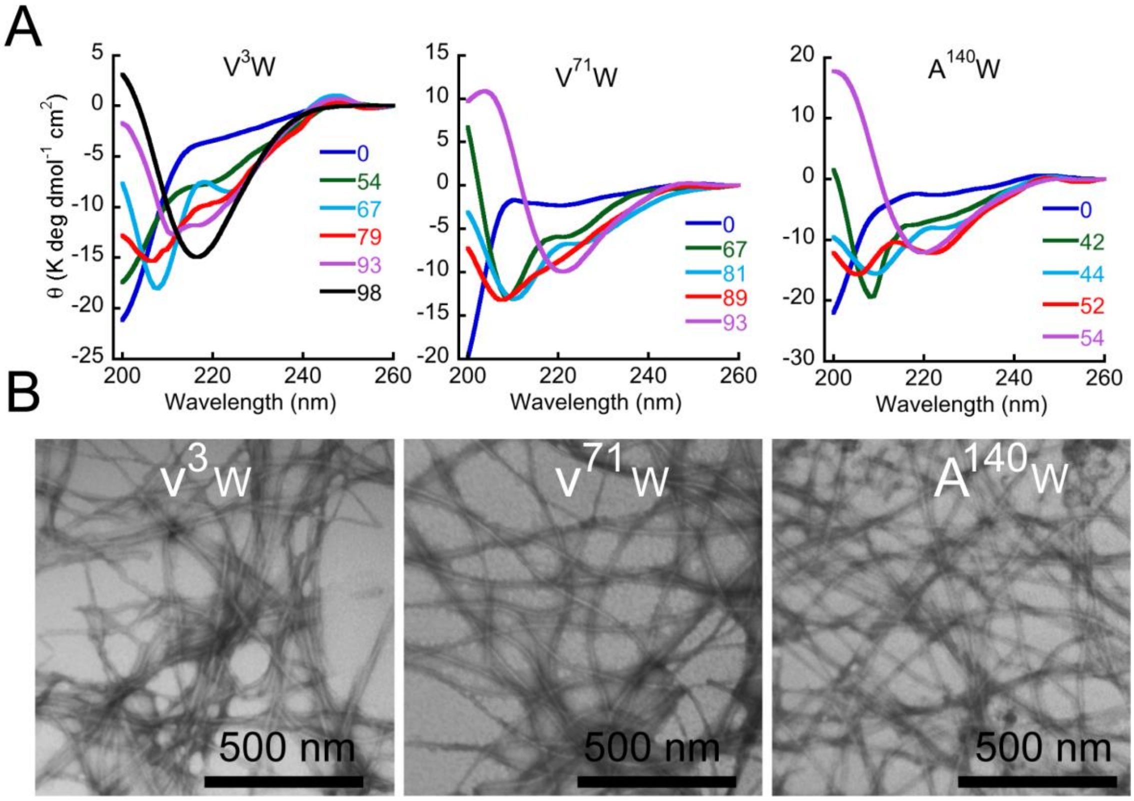

Supplementary Figure 10.

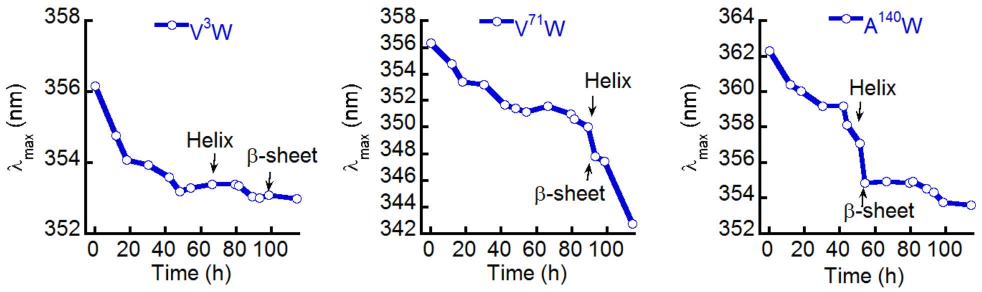

Supplementary Figure 11.

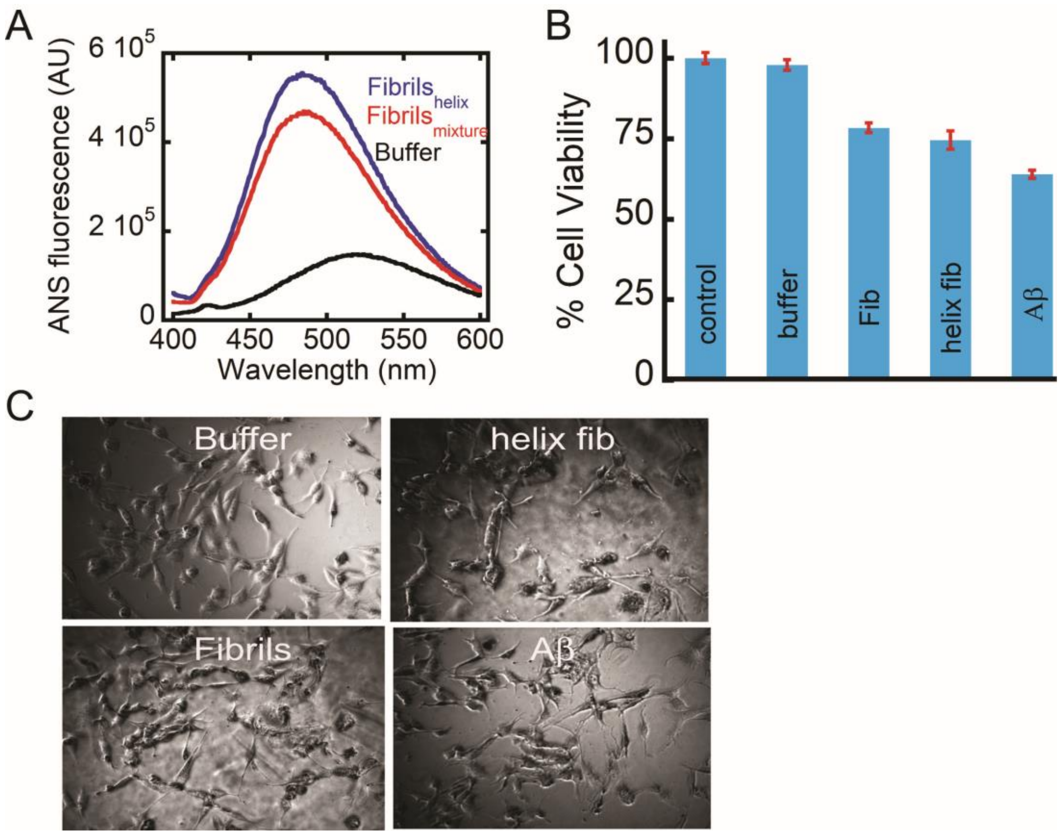

Supplementary Figure 12.

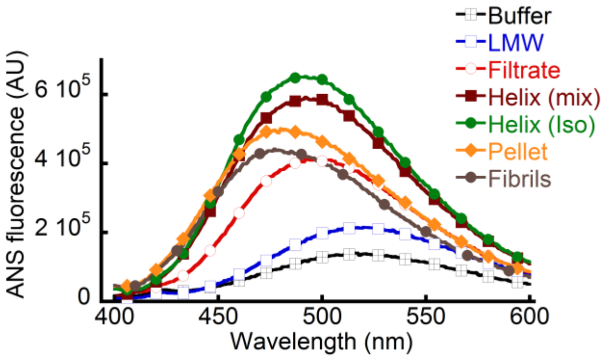

Supplementary Figure 13.

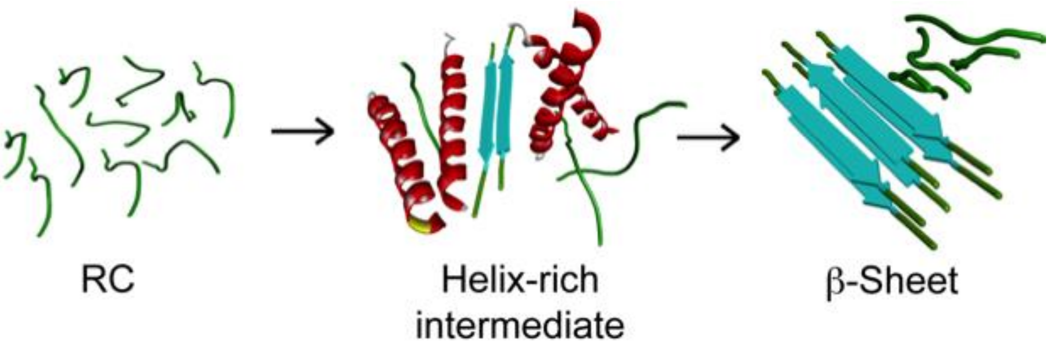

Supplement: Supplementary Information — Structure based aggregation studies reveal the presence of helix-rich intermediate during α-Synuclein aggregation [file srep09228-s1.pdf]
